# Reduced nonverbal interpersonal synchrony in autism spectrum disorder independent of partner diagnosis: a motion energy study

**DOI:** 10.1186/s13229-019-0305-1

**Published:** 2020-02-03

**Authors:** A. L. Georgescu, S. Koeroglu, A. F de C Hamilton, K. Vogeley, C. M. Falter-Wagner, W. Tschacher

**Affiliations:** 1 Department of Psychology, Institute of Psychiatry, Psychology and Neuroscience, King’s College London, Guy’s Campus, Addison House, London, SE1 1UL UK; 2 Institute of Cognitive Neuroscience, University College London, 17 Queen Square, London, WC1N 3AR UK; 3 Department of Psychiatry and Psychotherapy, University Hospital of Cologne, Cologne, Germany; 4 Institute of Neurosciences and Medicine – Cognitive Neuroscience (INM-3), Research Center Jülich, Jülich, 52425 Germany; 5 Department of Psychiatry, Medical Faculty, LMU Munich, 80336 Munich, Germany; 6 University Hospital of Psychiatry and Psychotherapy, University of Bern, Bern, Switzerland

**Keywords:** Interpersonal synchrony, Interactional heterogeneity, Interpersonal coordination, Autism spectrum disorder, Social interaction, Motion energy analysis, Autism spectrum disorders, Interpersonal coordination, Interpersonal synchrony, Motion energy, Social interaction, Dyadic interactions, Conversation, Nonverbal behaviour

## Abstract

**Background:**

One of the main diagnostic features of individuals with autism spectrum disorders is nonverbal behaviour difficulties during naturalistic social interactions. The ‘Interactional Heterogeneity Hypothesis’ of ASD proposes that the degree to which individuals share a common ground substantially influences their ability to achieve smooth social interactions.

**Methods:**

To test this hypothesis, we filmed 29 autistic and 29 matched typically developed adults engaged in several conversational tasks. Windowed cross-lagged correlations were computed using the time series of motion energy of both individuals in a dyad. These coefficients were then compared across the three dyad types that were homo- or heterogenous with respect to diagnosis: pairs of two autistic individuals, two typically developed individuals or pairs of one autistic and one typically developed person.

**Results:**

We found that all dyad types achieved above-chance interpersonal synchrony, but that synchrony was more expressed in typical dyads compared to both autistic and mixed dyads.

**Limitations:**

The method presented here provides only one, albeit objective and robust, approach to explore synchrony. The methodological choices as well as the lack of consideration for other communication modalities may limit our interpretation of the findings. Moreover, the sample size is small with respect to exploring associations between synchrony and various outcome and social skill measures.

**Conclusions:**

The present results do not provide support for the Interactional Heterogeneity Hypothesis given that autistic individuals do not coordinate better when interacting with another autistic individual, compared to when interacting with a typical individual.

## Background

Individuals with autism spectrum disorder (ASD) are characterised by life-long difficulties in communication and reciprocal social interaction [1]. In particular, ASD has been associated with atypical social contingencies which include difficulties in coordinating attention [2] and interactive turn-taking [3, 4]. However, it has been proposed that such difficulties may be attributable to an ‘Interactional Heterogeneity’ across persons, rather than solely to individual failure [5–8]. This suggests that a breakdown in social interaction and mutual understanding can happen between people with very differing ways of processing and experiencing the world (ibid.). To date, it is still unclear whether an ‘interactional heterogeneity’ underlies these difficulties in social interaction in ASD.

One important characteristic of most social interactions is the unintentional coordination of various behaviours, such as heart rate, affect or vocal output [9]. In fact, one of the most investigated phenomena is that of the spontaneous and unintentional coordination of people’s moving bodies [10–16]. In this article, we will use the term interpersonal synchrony (IPS) to denote this type of coordination. It can include a broad range of nonverbal behaviours and ranges from simultaneous occurrence of behaviours to behaviours involving a short delay [17, 18]. IPS may underlie interaction success because it can promote effective turn-taking, as well as connectedness, trust and prosocial behaviour [11, 19], and it can even predict the success of a problem-solving exercise, negotiation or meeting [20, 21]. It has also been associated with various psychopathological conditions like schizophrenia [22, 23], social anxiety disorder [24] and borderline personality disorder [25]. We therefore argue for an in-depth investigation of IPS in ASD, in order to understand its contribution to the social interaction problems characteristic of the condition.

So far, the ability to coordinate movements with another person has been mostly studied in persons with ASD using paradigms that are highly rhythmic and therefore not directly related to naturalistic interactions [26, 27]. Reduced and more variable IPS between children or adolescents with ASD and an experimenter has mostly been observed with intentional coordination tasks, like a synchronous interpersonal hand-clapping task [28, 29], intentional synchronisation of a swinging pendulum [27], synchronised object tapping [29] or in a social motor coordination battery [28].

However, while such rhythmic or temporally stable behaviours do play an important role in IPS, there are many other potential sources of unintentional coordination that need to be taken into account if it comes to naturalistic interactions [30]. Real-world social interactions are seldom perfectly rhythmic but rather highly complex and unpredictable. The ability to coordinate with another person in a naturalistic setting seems to be impaired early on in ASD development [3, 31, 32]. In a recent investigation, Romero and colleagues [33] looked at whole-body IPS between children with ASD and a clinician during a conversational exchange. Interestingly, the study found that children with ASD achieved IPS with a clinician, that these movements were complex and that the complexity of the children’s movements matched that of the clinician. Importantly, however, the degree of bodily coordination was related to higher social cognitive ability. A similar study on children performing a test battery (including breaks) with an experimenter showed differences for head and hand movement IPS [34]. Like Romero and colleagues [33], this study was done in children, but it additionally included control dyads of typical children interacting with a clinician. Taken together, these studies show that individuals with ASD do achieve IPS, but they tend to do so to a lesser extent compared to typical participants.

It is important to understand what underlies such IPS difficulties in ASD. The understanding of psychopathology in an interpersonal context has long been present in psychiatry [35–38]. Several recent theoretical accounts have suggested that the difficulties with social interactions that are symptomatic of ASD are interactional problems instead of individual ones. Hanne De Jaegher [7, 39] suggests that, given that ASD individuals have a different embodiment (including intra-personal coordination and different sensory and perceptuo-motor skills, see also Gallagher [40]), this may lead to problems in interpersonal coordination. This can also make it difficult to find common ground with another, given that social meaning is negotiated by the interplay of the interaction process and the individuals engaged in it (“participatory sense-making”) [7, 39]. What Damien Milton [8] calls the “double-empathy problem” refers to the idea that individuals who have different ways of processing and experiencing the world will also have differing norms and expectations and would therefore find it difficult to empathise with each other (see also [41]). It is a “double” problem because both social actors involved in a social interaction experience it. Annika Hellendoorn highlights the importance of dissimilarity of ASD in terms of perceiving and sharing affordances in the (social) environment which may disrupt smooth social interactions [42]. The “cross-neurological theory of mind” account of Luke Beardon [43] suggests that perspective taking is relative to neurological states and that there is not one universally correct way to represent other people’s thoughts. Thus, social interaction difficulties often arise due to a lack of accommodation and acceptance of this neurodiversity. The “dialectical misattunement hypothesis” [5] understands ASD as a cumulation of wrongly attuned experiences between persons. These are understood as disturbances of the dynamic and reciprocal unfolding of an interaction across multiple time scales and can result in individuals developing divergent interactional styles. We further refer to the ‘Interactional Heterogeneity Hypothesis’ (IHH) of ASD to summarise these accounts that share a similar way of understanding social interaction difficulties in ASD. Importantly, however, on the flipside, this IHH would also suggest that those with similar experiences are more likely to achieve a smooth and successful interaction [8]. This suggests that social interactions between individuals with ASD might show an advantage over social interactions between individuals with and without ASD. To date, only two other studies have investigated the IHH to explain autistic social interaction difficulties [44, 45]; however, both of these studies investigate different aspects of social interaction difficulties and reach different conclusions. Wadge and colleagues [44] used a cleverly designed computerised task of a strategic interactive partner game and Crompton and colleagues [45] used diffusion chains of verbal information transfer in groups of eight participants.

In sum, research has rarely investigated IPS in naturalistic situations due to methodological challenges of capturing its complexities. A few studies mainly explored children or adolescent populations using highly rhythmic actions [26, 46] or interactions in a controlled and formal clinical setting [33, 34]. Furthermore, the IHH has received a lot of theoretical attention and very little empirical investigations. To this end, we will quantify IPS in natural interactions with individuals with ASD, and we will compare hetero- and homogeneous dyads. There were 9 heterogenous ‘mixed’ dyads, 10 homogenous ASD dyads and 10 homogenous ‘typical’ dyads interacting in five different scenarios, resulting in 144 videos. In line with the IHH we predict that the homogeneity of a dyad is conducive to a higher amount of IPS as an index of interaction quality and that ASD dyads will achieve similar levels of IPS to typical dyads. We further predict that homogenous dyads will achieve more favourable evaluations of the interaction and their partner. This would lend support to the IHH of social interaction difficulties in ASD.

## Methods

### Sample

A group of 29 individuals with ASD and a group of 29 matched typically developed control persons participated in this study (see Tables 1 and 2). Fluency in German was an inclusion criterion. The 29 ASD participants (17 males) were between 23 and 56 years of age (*M* = 42.76, SD = 9.79, see Table 1) and were diagnosed and recruited in the Autism Outpatient Clinic at the Department of Psychiatry of the University Hospital of Cologne in Germany. As part of a systematic assessment, the diagnoses were confirmed by clinical interviews according to ICD-10 criteria by two specialised clinical experts and were supplemented by extensive neuropsychological assessment. The sample included patients with the diagnoses Asperger syndrome/high-functioning autism with an at least average Full Scale IQ (FSIQ > 85, measured using Wechsler Adult Intelligence Scale, WAIS). Six of the ASD participants were taking psychotropic medications (1x Lamotrigin and Olanzapin, 1x Fluoxetin, 1x Clomipramin, 1x Escitalopram, 1x Venlafaxin and Methylphenidat, 1x Valproic Acid). It is important to note that medication and depression can affect the quantity of nonverbal behaviour produced and therefore also IPS [47, 48]. As depression is a common co-morbidity in ASD [49, 50], and because we did not find significant differences of motion energy between ASD participants with and without medication (*t*(13,133) = − 1.527, *p* = 0.150) or with and without BDI scores higher than 20 (i.e. moderate depression, *t*(15,037) = − 1343; *p* = 0.199), they were not excluded from the sample. The 29 typically developed participants (17 males) were between 25 and 56 years of age (*M* = 41.31, SD = 9.10, see Table 1) and were recruited online from the student and staff population at the University of Cologne and the University Hospital of Cologne, Germany. They reported no history of psychiatric or neurologic disorders, and no current use of any psychotropic medications. In order to avoid clinically significant autistic traits in the control sample, control participants were included only if scoring less than 26 on the autism quotient (AQ) [51, 52].

**Table 1:** Demographics and questionnaires table

Test	ASD (*n* = 29)	typical (*n* = 29)	Group comparison (*p* value)
Gender (m/f)	17/12	17/12	
Age	42.76 ± 9.79	41.31 ± 9.10	.562
AQ	42.45 ± 4.24	14.69 ± 4.64	.000
EQ	15.38 ± 7.89	50.37 ± 10.08	.000
SQ	42.62 ± 14.72	25.52 ± 10.31	.000
TAS20	64.66 ± 10	44.34 ± 11.34	.000
BDI	12.24 ± 10	5.52 ± 4.46	.002
WST	113.17 ± 11.05	111.54 ± 8.60	.536

*Note:* Mean values and the respective standard deviations are displayed

*ASD* autism spectrum disorder, *n* sample size, *AQ* Autism Spectrum Quotient, *EQ* Empathy Questionnaire, *SQ* Systemizing Questionnaire, *TAS20* 20-item Toronto Alexithymia Scale, *BDI* Beck Depression Inventory, *WST* German verbal IQ test

**Table 2:** Dyad composition and matching

Test	ASD (*n* = 10)	TYPICAL (*n* = 10)	MIXED (*n* = 9)	Group comparison (*p* value)
Gender (m/f)	6/4	6/4	5/4	
Age avg	43.45 ± 9.65	41.80 ± 8.86	40.72 ± 10.45	.825
Age diff	2.70 ± 1.57	2.60 ± 1.58	2.11 ± 1.54	.688
AQ avg	43.05 ± 2.01	14.90 ± 3.93	27.67 ± 3.60	.000
AQ diff	3.70 ± 2.91	4.20 ± 3.82	26.89 ± 8.08	.000
EQ avg	15.85 ± 6.99	51.55 ± 7.81	31.06 ± 3.89	.000
EQ diff	7.30 ± 6.40	14.70 ± 10.71	33.44 ± 7.33	.000
SQ avg	44.15 ± 9.73	25.75 ± 8.16	32.11 ± 10.88	.001
SQ diff	18.30 ± 11.36	12.30 ± 10.90	16.67 ± 7.16	.403
TAS20 avg	66.30 ± 7.23	44.60 ± 10.32	52.39 ± 7.38	.000
TAS20 diff	10.40 ± 8.50	11.00 ± 12.14	17.22 ± 9.50	.295
BDI avg	12.55 ± 6.04	5.15 ± 3.54	8.94 ± 4.94	.010
BDI diff	14.10 ± 11.55	4.10 ± 3.03	7.89 ± 6.97	.032
WST avg	113.55 ± 6.34	111.17 ± 6.74	111.33 ± 6.91	.684
WST diff	15.90 ± 8.71	11.00 ± 6.18	10.22 ± 5.26	.173

*Note:* Mean values and the respective standard deviations are displayed

*ASD* autism spectrum disorder dyads, T*YPICAL* typical dyads, *MIXED* mixed dyads, *n* sample size, *avg* average dyad value, calculated from the average score of both individuals of a dyad; *diff* difference dyad value, calculated from the average score of both individuals of a dyad; *AQ* Autism Spectrum Quotient, *EQ* Empathy Questionnaire, *SQ* Systemizing Questionnaire, *TAS20* 20-item Toronto Alexithymia Scale, *BDI* Beck Depression Inventory, *WST* German verbal IQ

For matching purposes, intelligence in both diagnostic groups was assessed using the German multiple-choice verbal IQ test (“Wortschatztest”, WST [53];. Known to provide a valid and time-effective estimate of intelligence [53–55]. Furthermore, a series of questionnaires were filled out, in order to better describe the samples: The autism questionnaire (AQ, [51]), the Empathising Quotient (EQ, [56]), the Systemizing Quotient (SQ, [57]). Each participant also completed questionnaires assessing comorbidities, namely, the Becks Depression Inventory (BDI, [58, 59]) and the 20-Item-Toronto Alexithymia Scale [60, 61]. Table 1 describes the ASD and typical samples and shows, consistent with the clinical diagnoses, significant differences between the two groups in the scores of the AQ, EQ and SQ. Consistent with the two common ASD comorbidities [49, 50, 62, 63], significant differences between the two groups were also found in the BDI and the TAS20 scores (see Table 1).

Participants were assigned to one of three types of dyads, made up of either two ASD individuals (ASD), two typically developed individuals (typical) or mixed dyads of an ASD and a typical individual (mixed, see Table 2). There were 10 ASD dyads, 10 TYPICAL dyads and 9 MIXED dyads. To achieve optimal matching, dyad assignment rules were that interacting partners would have the same sex, be not more than +/− 5 years apart in age and +/− 2 SD in IQ. Table 2 shows average and difference dyad scores on all the questionnaires and tests to better describe the three dyad types.

Participants were naïve with respect to the purpose of the study or the diagnostic status of their partner. Instead, participants were informed that the experiment sought to analyse processes taking place in verbal conversations between unacquainted persons. The video and audio recording of interactions was explained as being a prerequisite for subsequent evaluation of discussion performance. The audio-visual recording of interactions was openly declared in the recruitment description. Written informed consent was obtained from all participants in accordance with the Declaration of Helsinki (2013). All participants received a monetary compensation for their participation of 40 Euro and were debriefed at the end. The study was conducted with approval of the local ethics committee of the Medical Faculty of the University of Cologne.

### Video setup and interaction scenarios

The project consisted of dyadic interactions between previously unacquainted persons of the same sex. When participants arrived at the lab, the experimenter welcomed them and briefly introduced them to each other and explained the sequence of events. Each person individually completed the battery of neuropsychological measures and questionnaires prior to the interaction sequences. The interactions were conducted in a room with standardised and stable artificial lighting and seating arrangements. All conversations were recorded using a high-definition video camera (Panasonic DV C Pro HD P2), mounted on a tripod 320 cm away from the chairs which were 60 cm apart from each other (floor markings ensured standard placement). All interactions lasted for 5 min, and the order of the interaction scenarios was as follows: An ice-breaker task (desert island), a cooperative and a competitive debate, two fun tasks (meal planning and knock-knock jokes) and a role play.

First, there was an ice-breaker task, where participants were asked to engage in a 5-min-long unstructured conversation with respect to which five items they would take with them to a desert island. This was followed by two verbal debates, one cooperative and one competitive on randomly assigned social and political topics of general interest drawn form an urn of eight topics [64, 65]. Participants of a dyad were provided with one of two different written lists of specific arguments fitting these topics, which they could read in a preparation period of 2 min prior to the interaction. One instruction encouraged cooperation and one encouraged competition. The cooperation instruction was to develop a shared position with the strongest arguments from the lists and imagine that they would have to persuade a third party. The competition instruction was to argue against the position of the interaction partner; whereby one participant received a longer list of five strong arguments, and the other a list of two weaker arguments. The sequence of instructions was randomised and balanced, with 50% of dyads receiving cooperation first and competition after and 50% receiving them the other way around. The next interaction was a ‘fun task’ adapted from [66]. The instruction was to design a five-course meal composed of dishes and drinks that both participants dislike. This was followed by a German knock-knock joke telling task [67, 68]. Each participant was asked to memorise three previously given jokes. Finally, a 5-min role play took place where one participant was randomly assigned the role of the boss and the other of the employee of a big insurance company. Participants had 2 min time, prior to the interaction, to read the context of the story. They were told to enact a meeting, where a boss and an employee negotiate a situation [69].

### Evaluation measures

After each interaction task, participants were required to fill out a post-test questionnaire, where they appraised the “positiveness” of each of their interactions on three items on a 6-point scale. These items were as follows: (1) How easy was the task? (2) How pleasant was the interaction? and (3) How likeable was their interaction partner? Higher scores reflected a more positive evaluation of the partner and the interaction.

### Data analyses

#### Video analysis and frame differencing approach

##### Video selection and pre-processing

The entire knock-knock joke video category was excluded from analyses because no dyad was able to complete the task and memorise the jokes to the standard required. We therefore proceeded to analyse all the other videos from five different interactions: ice-breaker, cooperative and competitive debates, meal-planning and role play. This resulted in 144 videos: 50 for the ASD group, 50 for the TYPICAL group and 44 for the MIXED group. One further video was excluded since participants did not understand the instruction: the ice-breaker task in one of the MIXED dyads. Here, we used an imputation method, whereby we replaced the data of this one dyad with the average motion energy and IPS values of that task across all dyad types.

##### Time-series extraction using Motion Energy Analysis

**Fig. 1:**
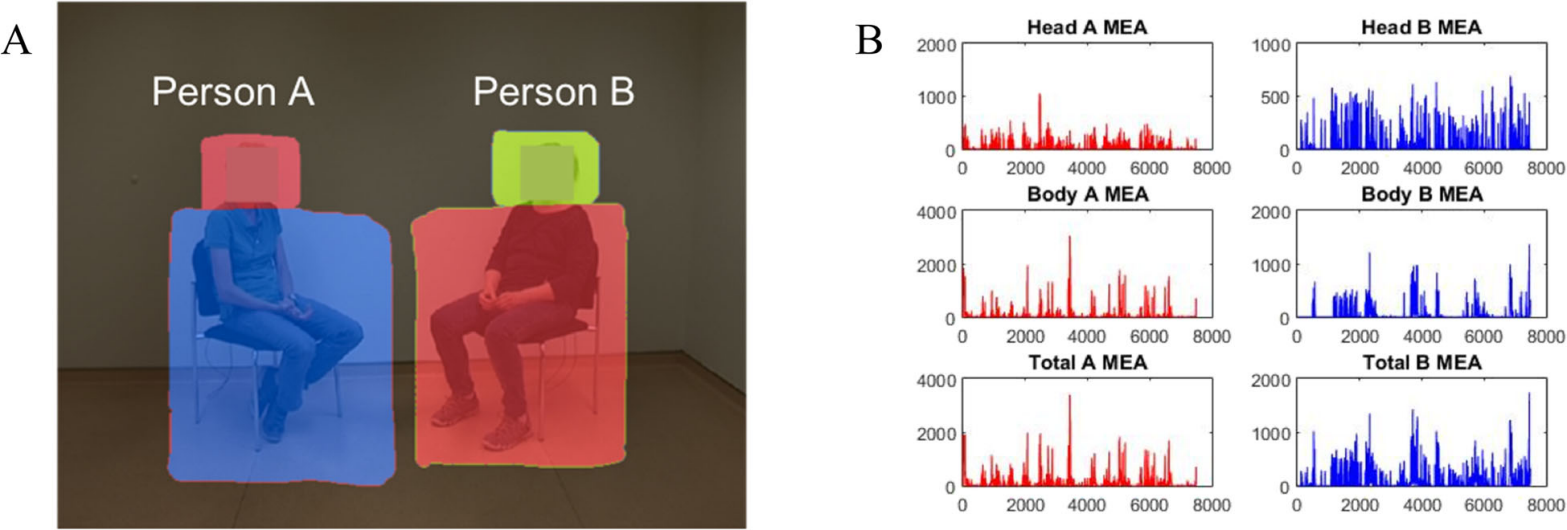
Motion energy analysis. **a** Top row, still frame of video showing a dyad with the ROIs as boxes of different colours. **b** Time series of individual motion energies (*y*-axis: motion energy values; x-axis: time in frames (rate: 25 frames per second)

Motion energy analysis is an objective frame-differencing method to determine changes in movement from videos [14, 70]. It is automated to continuously monitor the number of pixels changing in pre-defined regions of interest (see www.psync.ch for details). We selected two regions of interest (ROI) for each participant, covering (1) the head and (2) the rest of the body including the legs (Fig. 1). These were drawn individually for each video using the tools provided by the motion energy analysis software user interface. Absolute changes in grayscale values in these ROIs were detected and separately recorded as numerical streams of data, thus generating two continuous time series measuring the amount of movement in the head and the body region of each interactant. Because the ROIs are mutually exclusive and additive, we also computed a sum of the two ROIs to achieve total full-body motion energy data from each participant.

##### Synchrony and pseudo-synchrony computation using windowed cross-lagged correlation of time series

The quantification of synchrony was achieved by using windowed cross-lagged correlations of the motion energy time series of both participants in each dyad in every 5-min interaction [70–73]. This correlation approach [72] yields a measure of the similarity of two time-series, as a function of the displacement ('lag') of one relative to the other. Time-lags of up to +/− 5 s (i.e. in both directions) are applied in steps of 0.1 s, i.e. the time series are shifted by 0.1 s and then correlated. This procedure is repeated until all 101 time lags are covered (50 lags up to the maximum lag of 5 s in each direction, plus 1 correlation at lag zero). The cross-correlations were performed separately in all segments of 30 s duration of a 5-min interaction, in order to take into account the non-stationary nature of movement behaviours [65]. Cross-correlations were then transformed (Fisher’s *Z*), and their absolute values were aggregated over the entire interaction, yielding one global value of synchrony for each of the five interactions of each dyad. The use of absolute values means that both positive and negative cross-correlations contributed positively to the 5-min synchrony measure, so that anti-phase correlations also contributed to IPS. These values were used as dependent variables for the main analysis. In order to evaluate the significance of synchrony values, a control for coincidental synchrony is needed. To control for coincidental synchrony (e.g. [65]), we computed 90 surrogate interactions for each genuine one and then compared these to each other. To compute surrogate interactions, we shuffled the time series of each genuine dyad segment-wise. Hence, in a surrogate time series, movement segments of person A are aligned with movement segments of person B that never actually occurred at the same time. This procedure kept the time structure of the real data intact but only permuted the temporal location of the 30 s segments. Synchrony in all surrogate interactions (i.e. pseudo-IPS) was finally calculated identically to the synchrony of the original data as described above. This process yielded a distribution of pseudo-IPS values for each original dyad.

#### Statistical analyses

All analyses were done for the head, the body and total ROI. Because of (1) several instances of region-crossing (e.g. face touching, where the movement from the body ROI enters the head ROI), (2) non-standardisation of motion energy values to account for different sizes of the manually drawn head and body ROIs and (3) because the head and body ROIs are mutually exclusive and additive, we consider the total ROI to be the most robust dependent variable. Importantly, we did not find a significant interaction between our effects of interest and ROI (see Additional file 1), we therefore only report here the results from the total ROI. We report the separate head and body ROI results, for the sake of completeness, in Additional file 1.

For statistical analysis, we used JASP Version 0.10.1 [74]. To investigate motion quantity differences, the dependent variables were the averages of the motion energy time series of each person in each dyad for each task. To investigate IPS, the dependent variables were the average standardised cross-correlation coefficients for each dyad in each task. To demonstrate that IPS was significantly present at an above-chance level in all groups, irrespective of manipulation, we checked if the genuine IPS value of each original dyad came from the same distribution of pseudo-IPS values derived from the shuffled surrogate dataset by means of a *Z*-test. Analyses of variance (ANOVA) were performed to test for differences between dyad types in terms of IPS and the evaluation ratings, please see results section for details). If Mauchly’s test indicated that the assumption of sphericity was not fulfilled, degrees of freedom were corrected using the Greenhouse–Geisser estimates of sphericity. Holm-corrected post hoc tests were performed to better characterise the nature of the significant main effects. All effects are reported as significant at *p* < .05. Bayes factors were added using JASP, to aid the interpretation of results. We used the guidelines proposed by Jeffreys [75] for interpreting BF10.

## Results

### Evaluation measures

We investigated whether individual ratings of (1) how easy the task was, (2) how pleasant the interaction was and (3) how likeable the partner was differed significantly depending on the dyad type that the individual was part of. For each of these dependent variables, we ran a two-way repeated measures ANOVA with TASK as a within-subject factor (island, cooperative debate, competitive debate, meal planning, role play) and GROUP (ASD, MIXED, TYPICAL) as a between subject factor. We found significant main effects of TASK in all dependent variables (detailed results can be found in Additional file 1—evaluation), and for the pleasantness of the interaction rating, we found a significant main effect of group (*F*(2,55) = 3.336, *p* < .05, *η*_*p*_^2^ = 0.108) and a significant interaction effect for TASK x GROUP (F(5.827,160.248) = 2.683, *p* < .05, *η*_*p*_^2^ = 0.089). This suggests that overall, the rating of the interactions with respect to pleasantness was similar across groups but that individuals from the TYPICAL dyads rated the island and cooperative interactions as significantly more pleasant compared to individuals from the other two dyad types. Exploratory correlation analyses showed no significant relationships and are reported for reasons of completeness in Additional file 1.

### Movement quantity

**Fig. 2:**
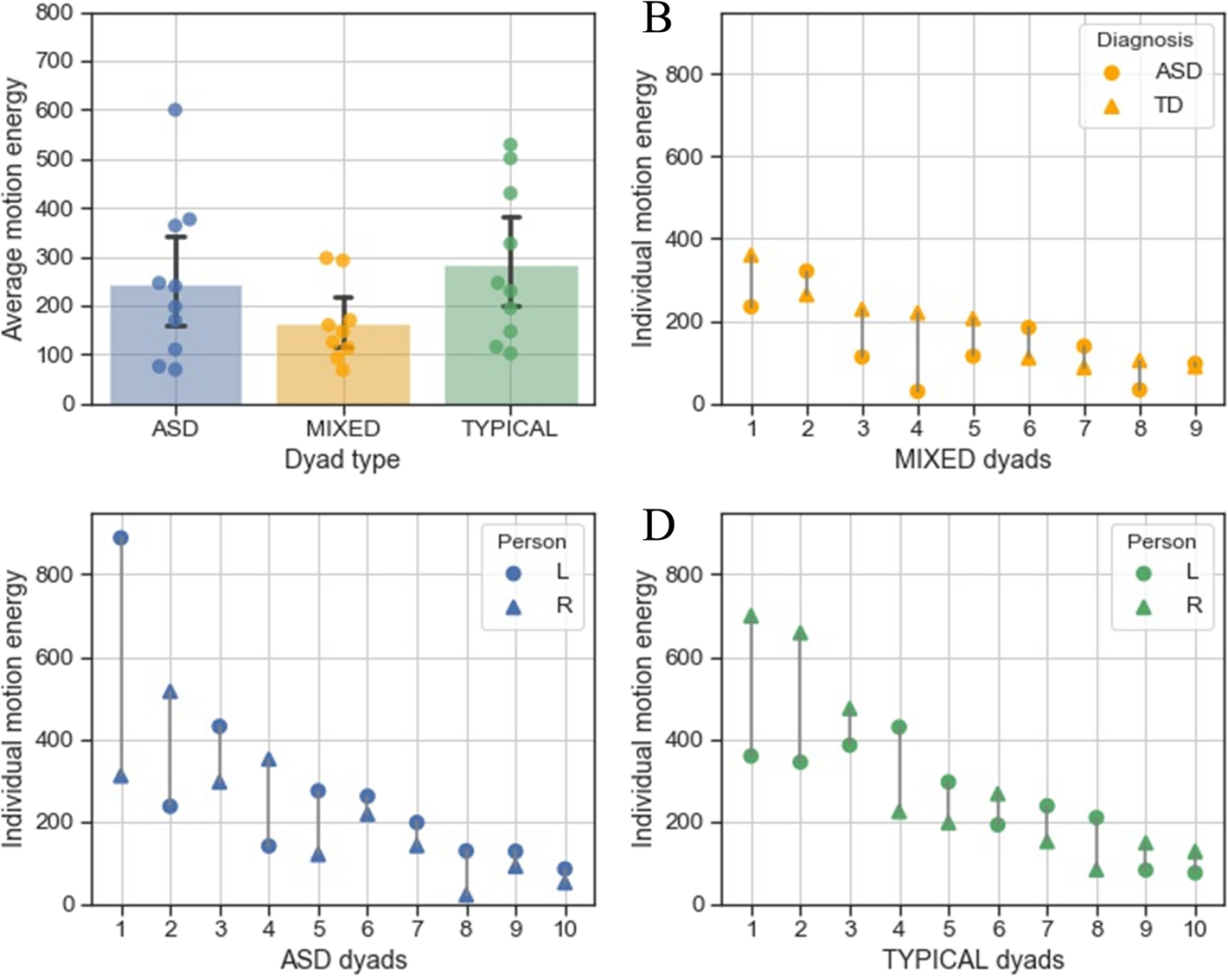
**a** Differences in average motion energy in three different dyad types (10 ASD dyads, 10 TYPICAL dyads, 9 MIXED dyads). Error bars are 95% confidence intervals, created with 1000 bootstrap samples; **b**–**d** Motion energy contribution of each partner across dyad types. *L* sitting on the left, *R* sitting on the right

We examined whether dyads differed with respect to motion energy. This was important because any differences between groups that we would have found here, would have cautioned our interpretation of the findings on IPS. We performed a two-way 5 × 3 ANOVA, defining TASK (island, cooperative debate, competitive debate, meal planning, role play) as within-subject factor and DYAD TYPE (ASD, TYPICAL, MIXED) as between-subject variable and the average motion energy dyad score as dependent variable. We found a main effect of TASK, *F*(3.121,171.669) = 3.926, *p* < .05, *η*_*p*_^2^ = 0.067. Post hoc tests revealed that the meal planning task resulted in significantly more average dyad motion energy compared to the island task (mean difference = 65.196, *t* = 3.816, *p*_Holm_ < .05, *d* = 0.501). There were no significant differences between dyad types (*F*(2,55) = 2.477, *p* = .093, BF_10_ = 0.867, equivalent to anecdotal evidence for no effect see Fig. 2a). This suggests that the different dyad categories moved on average to a similar degree. There was also no significant interaction between TASK and DYAD TYPE (*F*(6.242,171.669) = 0.934, *p* = .475).

### IPS versus pseudo-synchrony

The genuine IPS value of each dyad was computed as the absolute Fischer’s *Z* transformed cross-correlation values that were aggregated over the entire length of each interaction. Because the interaction between task and dyad type was not significant, we also averaged these values over all tasks for each dyad to attain the following mean *z* values for genuine synchrony: 0.49 (ASD), 0.74 (TYPICAL), and 0.8 (MIXED). These mean *z* values are identical to effect sizes (Cohen’s *d*), i.e., they demonstrate moderate to strong effects for synchrony against surrogate controls.

### IPS across dyad types

**Fig. 3:**
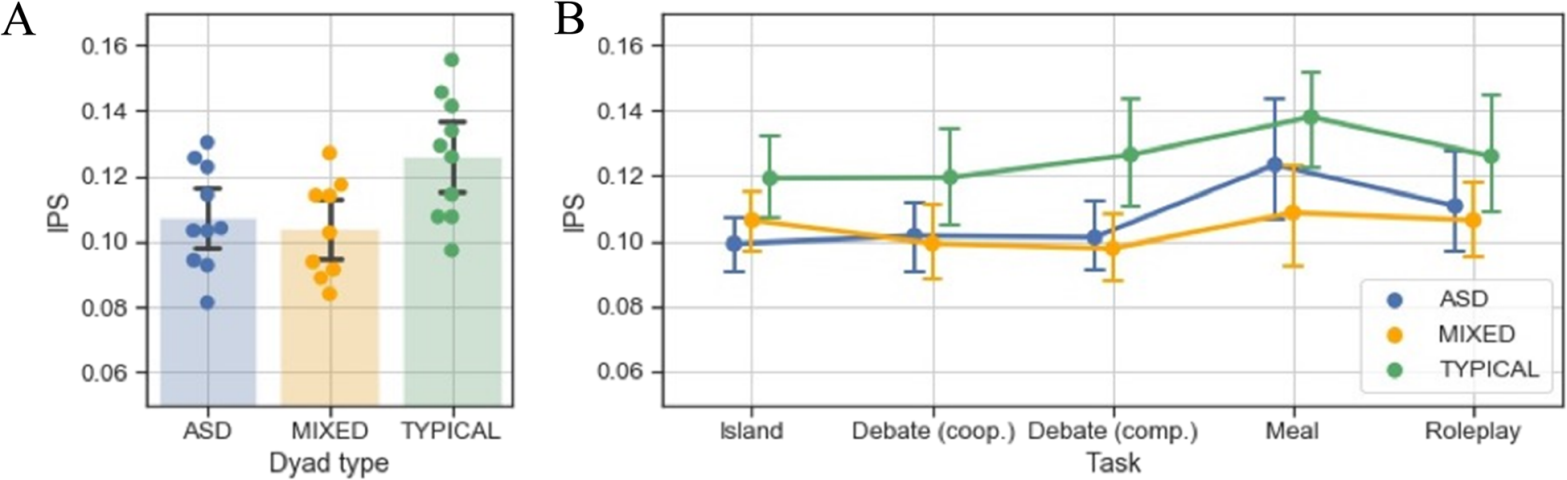
Interpersonal synchrony. **a** Main effect of dyad type. Average IPS aggregated over task type. Points represent individual dyads. **b** Differences in IPS between dyad types across tasks. Error bars represent 95% confidence intervals, created with 1000 bootstrap samples

To investigate IPS differences across groups, we performed a two-way ANOVA, defining TASK (island, cooperative debate, competitive debate, meal planning, role play) and DYAD TYPE (ASD, TYPICAL, MIXED) as a between-subject variable and IPS score as the dependent variable. There was a significant main effect of TASK on IPS, *F*(4,104) = 4.086, *p* < .05, *η*_*p*_^2^ = 0.136. Post hoc tests revealed that the meal planning task resulted in significantly more IPS than the cooperative debate (mean difference = 0.017, *t* = 3.338, *p*_Holm_ < .05, *d* = 0.620) and the island task (mean difference = 0.016, *t* = 3.098, *p* < .05, *d* = 0.575). There was a main effect of DYAD TYPE on IPS, *F*(2,26) = 4.955, *p* < .05, *η*_*p*_^2^ = .276, BF_10_ = 4.119 (see Fig. 3a). The Bayes factor for this test indicates that the data are 4.119 times more likely to be observed under the alternative hypothesis, in other words, it indicates substantial evidence in favour of the effect. Post hoc tests revealed that TYPICAL significantly differ from MIXED (mean difference = 0.022, *t* = 2.889, *p*_Holm_ < .05, *d* = 0.537, BF_10_ = 1327.480, equivalent to decisive evidence for the effect) and ASD (mean difference = 0.019, *t* = 2.498 *p*_Holm_ < .005, *d* = 0.464, BF_10_ = 72.315, equivalent to very strong evidence for the effect), but ASD did not differ significantly from MIXED (mean difference = 0.004, *t* = 0.458, *p*_Holm_ = .651, *d* = 0.085, BF_10_ = 0.286, equivalent to substantial evidence for no effect). Importantly, there was no interaction between TASK and DYAD TYPE, *F*(8,104) = 0.599, *p* = .777 (see Fig. 3b).

## Discussion

The present study shows whole-body IPS differences between typical dyads and dyads under participation of at least one (or two) individuals with ASD in a conversational setting. It extends previous findings on reduced IPS in ASD by looking at an adult population in an informal and naturalistic setting. Importantly, the study is not consistent with the IHH of autism, as results show no IPS advantage to a person with ASD having an interaction partner with the same diagnosis and no differences in evaluations between groups. In the following, we first discuss the overall motion energy findings and finally, the IPS findings and some limitations of the present study.

### Similar movement quantity for ASD and TYPICAL and across dyad types

Before interpreting the results regarding how much people can synchronise, we have to investigate the results with respect to the amount of motion energy that is produced. If there are systematic differences between groups in terms of how much dyads move in general, then this could also lead to systematic differences in IPS and ultimately affect how we interpret the IPS findings.

Indeed, scoring criteria for various diagnostic measures suggest that individuals with ASD produce less nonverbal behaviours than their typically developing peers (for a review, see [76]). If individual differences exist, one would expect also dyad type differences. Importantly, however, most empirical research findings have failed to find group differences between ASD and typical individuals in movement quantity as measured by amount of gestures [76–78]. Following that, in the present study, we did not find a significant difference in average dyad motion energy across dyad types (see Fig. 2). This may suggest that, on average, participants moved to a similar extent, irrespective of their own or their partner’s diagnostic condition. Nevertheless, Bayes factors suggest anecdotal evidence for the null effect. Given the small sample size and the study being underpowered, this needs to be interpreted with caution. Taken together, we conclude that the differences in IPS discussed below cannot be attributed to mere differences in terms of movement quantity and have plotted individual movement quantity for each dyad in Fig. 2b–d.

### Dyads with autistic participants show reduced IPS

Our finding that TYPICAL dyads achieved more IPS compared to MIXED dyads is in concert with the idea that social interaction should be more challenging and less well coordinated when there is a mismatch of interactional styles between two partners [5, 8, 39]. Such interactional styles may emerge due to differing processing styles and experiences of the world. Importantly, however, the IHH would also imply that ASD dyads may be able to achieve just as much IPS as TYPICAL dyads. This would be since they are homogenous with respect to the two partners’ diagnostic condition and hence more similar in interactional style. Thus, the present results do not support this hypothesis, because we find that all dyads with ASD individuals in their composition (MIXED and ASD) achieve less IPS compared to TYPICAL dyads. This is in line with recent research that shows greater conceptual misalignment when ASD and MIXED dyads are faced with ambiguous problems in a more explicit and strategic computerised game task [44]. Although the current study was not specifically designed to disentangle between them, in the following, we consider four lines of research that may explain why individuals with ASD achieve less IPS in social interactions.

One possible explanation for the present findings is related to the motor difficulties that ASD individuals experience. A prominent motor atypicality included in the diagnostic criteria for ASD is the presence of repetitive behaviours or stereotypies [1]. Moreover, research has found that individuals with ASD have delays in their motor development and that they move differently compared to typically developing individuals, with an atypical gait, postural control and upper limb movements as well as an impaired fine motor control [28, 79–86]. Fournier and colleagues [81] have reviewed 41 studies on motor abilities in ASD, in both infant as well as adult samples. They conclude that ASD individuals are characterised by weaker motor performance compared to typical controls, irrespective of symptom severity. This may be linked to differing cerebellar functions [83, 87]. Importantly, ASD individuals are also more variable in motor performance (e.g. [25, 85, 87]) and planning [88, 89]. Following Dowd and colleagues [90], we argue that motor function is important because interpersonal interactions and communication rely on it for execution. For the present findings, we could argue that atypical movements and movement variability make it difficult for coordination between individuals to occur. Unfortunately, we did not directly assess motor abilities in the present sample. However, Fitzpatrick and colleagues [46] have found that, while IPS was associated with ASD severity, it was not fully explained by motor problems. In particular, only the more rhythmic types of IPS were related to motor ability. Such rhythmic interactions involve a very predictable structure, but this is unlike the conversations used in the present study. In a similar line, Noel and colleagues [34] highlight that motor ability is not sufficient to account for the differences they found in IPS between ASD and typical individuals and that the difference in achieved IPS between ASD and typical individuals was uncoupled from differences in movement complexity.

An alternative explanation may be related to the perception side rather than the production side of nonverbal behaviour. Research has shown that individuals with ASD have atypical perception and attention [91–94], as well as more specifically difficulties interpreting nonverbal cues (for a review, see [95]). Also, the degree to which nonverbal information contributes to social processing in ASD is significantly lower than in control participants [96, 97]. For example, Georgescu and colleagues [96] showed that persons with ASD do not use subtle aspects of gaze duration to form impressions of others in an ambiguous context. Kuzmanovic and colleagues [97] found that, although ASD participants could evaluate nonverbal behaviours in isolation (e.g. an observed person leans forward with a smile), they showed a reduced sensitivity to nonverbal cues when this information conflicted with verbal information. It is then possible that individuals with ASD rely more strongly on explicit information. It is impossible to test this with the current design, yet future research should investigate whether interpersonal coordination may be achieved in ASD dyads using a different channel or modality (e.g. via the more explicit verbal channel).

ASD has also been associated with atypical time processing. Interviews have shown that individuals with ASD tend to rely on routines and repetitive behaviours to help the structuring of their subjective time experience [98, 99]. Moreover, empirical findings found that individuals with ASD tend to perceive time atypically ranging from impaired interval timing to intact or increased temporal event structure coding and that this correlates with nonverbal communication difficulty (e.g. [100–103]). Given the heterogeneity of the autistic phenotype and assuming a more variable temporal processing style in ASD individuals, it may be that in both ASD and MIXED dyads neither partner’s temporal style can reach close enough to the other in order to coordinate. This is similar to the idea of coupled oscillators in physics, whose frequency ranges need to be similar for them to entrain and synchronise [39, 104]).

The predictive coding account of ASD suggests that expectations about the precision of sensory inputs, relative to the precision of prior experiences may be essential in coordinating the interplay between perception, action, and social behaviour [5, 105, 106]. Noel and colleagues [34] find recent evidence that the association between multisensory perceptual ability (i.e. acuity of the temporal binding window) and IPS is missing in ASD children, but not in typically developing children. This would suggest that ASD children do not make use of sensory evidence in achieving coordination with another person.

To put our findings in the context of research investigating the IHH, our findings are in concert with findings from Wadge and colleagues [44] who find that ASD dyads have difficulties in a nonverbal task just as MIXED dyads have (not involving bodily movements but a computerised strategic interactive partner game). On the other hand, they contradict findings by Crompton and colleagues [45]. These authors find that, on an explicit verbal task of recounting a story in a diffusion chain of 8 individuals, ASD and typical groups were equally good at retaining the details of the story, whereas the MIXED group showed a steeper decline in detail retention. It is therefore important to highlight, given the difficulties of individuals with ASD with implicit but not explicit processing [107] that there may be differences in terms of coordination ability in ASD, depending on the domain of investigation.

## Limitations

### Sample limitations

A common problem in dyadic research is a small sample size [73]. A study’s sample size is halved given that all participants are studied in pairs and the unit of analysis is the dyad rather than the individual. While the sample size in the current study may seem small, it is comparable to or even higher than other sample sizes in both autism research [107, 108] and IPS research [34, 71, 109–111]. Nevertheless, it is important to note that, in order to compare between groups with sufficient power and to investigate associations with other variables more extensively, the present results would need to be replicated with a larger sample.

Further, it is important to note that the present ASD sample included adult individuals with the diagnoses Asperger syndrome/high-functioning autism with an at least average Full Scale IQ. The tasks in this study required the ability to use words to communicate and engage in conversations on a variety of topics. Even though our sample was restricted to a subgroup of autistic individuals, our findings support previous research which involved work on children on the broader spectrum, showing that individuals with autism can synchronise with others but they do so to a lesser extent, compared to typical individuals. Nevertheless, further research is needed to replicate the results of the present design and dyad type differences across the entire autistic spectrum and across development.

Furthermore, ASD individuals often score high on social anxiety measures and report higher sensibility to stress and performance anxiety [112]. The participation in the current study was advertised to involve several interactions between strangers. Therefore, we need to consider that the ASD individuals who volunteered might have either been already quite good at social interactions or at least not intimidated by them. Alternatively, they might have found social interactions particularly challenging and by volunteering, they would have been actively searching for opportunities to practise their social skills. In either case, the present sample might have suffered from a selection bias in the ASD group.

Finally, whereas in the TYPICAL dyads, we can assume that the nonverbal behaviour rules are shared between interaction partners, and in the MIXED dyads, this is clearly not the case, the ASD dyads represent a special case. While in the present study, ASD dyads are matched with respect to diagnosis, given the inherent phenotypic heterogeneity of ASD, it is more likely that atypicalities in ASD behaviour are idiosyncratic rather than shared. Some examples mentioned before in the manuscript relate to the variability within the ASD clinical presentation in terms of motor performance and temporal processing style. This would render what we considered homogenous dyads, in effect, heterogenous. This has important implications for the IHH in autism research. If we are to assume that the basis for the IHH is the ability to establish a common ground between interaction partners, and that this may influence the level of IPS achieved, it is then important to consider that ASD dyads may need to be more closely matched on relevant factors.

### Methodological limitations

The approach we use to quantify movement in dyads involves a frame differencing method called motion energy analysis and windowed cross-lagged correlations. Although very useful and easy to use, there are several limitations related to these methods.

Compared to other methods of movement tracking (e.g. motion capture equipment), motion energy quantification cannot track moving ROIs and it loses detail (e.g. movement direction and velocity) [73]. As opposed to motion energy analysis, more sophisticated motion capture can track single joints, and thus, the need for moving ROI tracking in video material is eliminated. On the other hand, just like motion energy analysis, motion capture does not take into account qualitative features such as the valence, type and/or meaning of participants’ nonverbal behaviour (e.g., approaching vs. disengaging postures). The use of traditional video annotation or state-of-the-art computer vision tools that can extract and categorise poses and expressions can be used as complementary tools in such investigations to help shed more light on the association of these qualitative features of nonverbal behaviour and their relation to IPS and its social outcomes. For example, once movement has been labelled using classic annotation and a coding scheme, this information can then be entered into a multivariate approach for the detection of the temporal structure of behaviour [113].

The result of the motion energy analysis can also depend on factors such as contrast or colours of the clothes, because these can affect greyscales and it is indicated to standardise this MEA investigations. In the present study, only a minority of participants did not wear dark clothing but the proportion of dark, bright or mixed clothed individuals did not differ between our experimental groups of interest (*χ*^2^ (4) = 4.94, *p* = .29).

Moreover, our approach is only one of many existing methods used to measure IPS. Schoenherr and colleagues [18] have recently reviewed several linear methods of time series analysis that can be used to quantify IPS and find that they address different aspects of coordination. In addition, nonlinear methods have been used to quantify how and the extent to which streams of information come to exhibit similar patterns in time [15, 114–116].

Further, synchrony may be computed on the basis of local trends rather than cross-correlations [117, 118]. Finally, IPS is a type of interpersonal coordination that can emerge not just in the time domain but also in the frequency domain [119]. In the frequency domain, IPS is represented as the amount of similarity between the spectral powers at which both partners move, at each frequency component (i.e. cross-spectral coherence). It would be important that future research considers all of these different aspects of IPS when investigating it.

In addition, IPS may emerge within and across different sensory modalities and timescales [12, 67, 115]. For example, the intensity of an infant’s movement matches the intensity of the mother’s speech [120]. Moreover, it has been shown that individuals may achieve synchrony and hence have positive interaction outcomes in lexical and nonverbal aspects of spoken language [121]. Paxton & Dale [73] highlight the importance of the relation between body movement, eye gaze and verbal behaviour and suggest that future research would gain a deeper understanding of the within- and cross-channel mechanisms of interpersonal coordination.

Finally, it is essential to relate IPS findings to a social outcome. Previous findings showed that IPS embodied both outcome and interpersonal variables of dyads [104]. Given the sample size (*n* = 29), the study is underpowered to interpret correlations between IPS and evaluation measures. For completeness, they are reported in Additional file 1. We have compared the dyad types with respect to the impressions that people get of the interaction and their partner after each task. We find no significant differences between individuals of the three different dyad types. However, this may also be due to the type of measures we employed: We used explicit ratings and it is very likely that more implicit measures are needed to investigate impressions that happen implicitly and outside of interactors’ awareness. In addition, process measures that can be administered pre- and post-interaction could be used in future studies [65].

## Conclusions

The current study helps gain a more complete picture of IPS in ASD in two ways: First, we show that there are IPS differences in informal, naturalistic interactions with adults with ASD without cognitive disabilities. Second, these IPS differences are unlikely to be the result of ASD and typical people being less able to coordinate with each other. Rather it is also equally reduced in homogenous ASD dyads. Thus, we find no support for the IHH using IPS as an index of interaction smoothness. This research opens up new questions with respect to further developing the IHH and the need to run more studies that help integrate the idea of the phenotypical heterogeneity of autism. This means that we may be able to understand Interactional Heterogeneity between (atypical vs typical development) and within (due to phenotypical heterogeneity) diagnostic conditions.

## Supplementary information

**Additional file 1.** Supplementary Materials – Synchrony (IPS). Supplementary Materials – Motion Energy. Supplementary Materials – Evaluation measures

## Data Availability

The datasets generated and/or analysed during the current study are not publicly available due to this being data from a sample that did not consent to their data being shared in any form, the raw data are not available to be shared. The data are available from the corresponding author on reasonable request.

## Abbreviations

AQ: Autism quotient
ASD: Autism spectrum disorder
BDI: Beck’s Depression Inventory
EQ: Empathy Questionnaire
IHH: Interactional Heterogeneity Hypothesis
IPS: Interpersonal synchrony
IQ: Intelligence quotient
ROI: Regions of interest
SQ: Systemizing Questionnaire
WST: “Wortschatztest” (German verbal intelligence test)
