# Supplementary material for: Reduced nonverbal interpersonal synchrony in autism spectrum disorder independent of partner diagnosis: a motion energy study
Source: Mol Autism. 2020 Feb 3;11:11. doi: 10.1186/s13229-019-0305-1 (PMC6998161; doi:10.1186/s13229-019-0305-1)

**Supplementary Analysis for the Evaluation Ratings**

Note:

ASD = ASD dyad type

TD = TYPICAL dyad type

MIXED = MIXED dyad type

group 3 = DYAD TYPE (between-subjects factor)

## How easy was the task?

Repeated Measures ANOVA

| **Within Subjects Effects** | | | | | | | | | | | | | | | |
| --- | --- | --- | --- | --- | --- | --- | --- | --- | --- | --- | --- | --- | --- | --- | --- |
|  | | **Sphericity Correction** | | **Sum of Squares** | | **df** | | **Mean Square** | | **F** | | **p** | | **η² _p_** | |
| Task |  | Greenhouse-Geisser |  | 84.777 | ᵃ | 3.427 | ᵃ | 24.741 | ᵃ | 16.932 | ᵃ | < .001 | ᵃ | 0.235 |  |
| Task ✻ group3 |  | Greenhouse-Geisser |  | 16.326 | ᵃ | 6.853 | ᵃ | 2.382 | ᵃ | 1.630 | ᵃ | 0.131 | ᵃ | 0.056 |  |
| Residual |  | Greenhouse-Geisser |  | 275.383 |  | 188.464 |  | 1.461 |  |  |  |  |  |  |  |
|  | | | | | | | | | | | | | | | |
| *Note.*  Type III Sum of Squares | | | | | | | | | | | | | | | |
| ᵃ Mauchly's test of sphericity indicates that the assumption of sphericity is violated (p < .05). | | | | | | | | | | | | | | | |

| **Between Subjects Effects** | | | | | | | | | | | | | |
| --- | --- | --- | --- | --- | --- | --- | --- | --- | --- | --- | --- | --- | --- |
|  | | **Sum of Squares** | | **df** | | **Mean Square** | | **F** | | **p** | | **η² _p_** | |
| group3 |  | 20.329 |  | 2 |  | 10.164 |  | 1.912 |  | 0.157 |  | 0.065 |  |
| Residual |  | 292.384 |  | 55 |  | 5.316 |  |  |  |  |  |  |  |
|  | | | | | | | | | | | | | |
| *Note.*  Type III Sum of Squares | | | | | | | | | | | | | |

Assumption Checks

| **Test of Sphericity** | | | | | | | | | | | | | | | |
| --- | --- | --- | --- | --- | --- | --- | --- | --- | --- | --- | --- | --- | --- | --- | --- |
|  | | **Mauchly's W** | | **Approx. Χ²** | | **df** | | **p** | | **Greenhouse-Geisser ε** | | **Huynh-Feldt ε** | | **Lower Bound ε** | |
| Task |  | 0.666 |  | 21.729 |  | 9 |  | 0.010 |  | 0.857 |  | 0.920 |  | 0.250 |  |
|  | | | | | | | | | | | | | | | |

| **Test for Equality of Variances (Levene's)** | | | | | | | | | |
| --- | --- | --- | --- | --- | --- | --- | --- | --- | --- |
|  | | **F** | | **df1** | | **df2** | | **p** | |
| Insel_schwierig |  | 4.222 |  | 2.000 |  | 55.000 |  | 0.020 |  |
| Koop_schwierig |  | 1.550 |  | 2.000 |  | 55.000 |  | 0.221 |  |
| Kompet_schwierig |  | 2.369 |  | 2.000 |  | 55.000 |  | 0.103 |  |
| Meal_schwierig |  | 1.553 |  | 2.000 |  | 55.000 |  | 0.221 |  |
| Roll_schwierig |  | 0.814 |  | 2.000 |  | 55.000 |  | 0.448 |  |
|  | | | | | | | | | |

Post Hoc Tests

| **Post Hoc Comparisons - Task** | | | | | | | | | | | | | | | | | |
| --- | --- | --- | --- | --- | --- | --- | --- | --- | --- | --- | --- | --- | --- | --- | --- | --- | --- |
|  | | | | | | **95% CI of Mean Difference** | | | |  | | | | | | | |
|  | |  | | **Mean Difference** | | **Lower** | | **Upper** | | **SE** | | **t** | | **Cohen's d** | | **p _holm_** | |
| comp |  | coop |  | -0.105 |  | -0.629 |  | 0.419 |  | 0.179 |  | -0.587 |  | -0.077 |  | 1.000 |  |
|  |  | island |  | -0.719 |  | -1.314 |  | -0.125 |  | 0.204 |  | -3.533 |  | -0.464 |  | 0.005 |  |
|  |  | meal |  | -1.482 |  | -2.046 |  | -0.919 |  | 0.193 |  | -7.686 |  | -1.009 |  | < .001 |  |
|  |  | roleplay |  | -0.228 |  | -0.919 |  | 0.463 |  | 0.237 |  | -0.964 |  | -0.127 |  | 1.000 |  |
| coop |  | island |  | -0.614 |  | -1.201 |  | -0.027 |  | 0.201 |  | -3.056 |  | -0.401 |  | 0.017 |  |
|  |  | meal |  | -1.377 |  | -2.005 |  | -0.750 |  | 0.215 |  | -6.409 |  | -0.842 |  | < .001 |  |
|  |  | roleplay |  | -0.123 |  | -0.805 |  | 0.560 |  | 0.234 |  | -0.525 |  | -0.069 |  | 1.000 |  |
| island |  | meal |  | -0.763 |  | -1.226 |  | -0.300 |  | 0.158 |  | -4.815 |  | -0.632 |  | < .001 |  |
|  |  | roleplay |  | 0.491 |  | -0.178 |  | 1.161 |  | 0.229 |  | 2.143 |  | 0.281 |  | 0.146 |  |
| meal |  | roleplay |  | 1.254 |  | 0.566 |  | 1.943 |  | 0.236 |  | 5.323 |  | 0.699 |  | < .001 |  |
|  | | | | | | | | | | | | | | | | | |
| *Note.*  Cohen's d does not correct for multiple comparisons. | | | | | | | | | | | | | | | | | |
| *Note.*  Bonferroni adjusted confidence intervals. | | | | | | | | | | | | | | | | | |

| **Descriptives** | | | | | | | | | |
| --- | --- | --- | --- | --- | --- | --- | --- | --- | --- |
| **Task** | | **group3** | | **Mean** | | **SD** | | **N** | |
| island |  | ASD |  | 3.578 |  | 1.445 |  | 20 |  |
|  |  | MIXED |  | 3.806 |  | 1.682 |  | 18 |  |
|  |  | TD |  | 4.775 |  | 1.045 |  | 20 |  |
| coop |  | ASD |  | 3.247 |  | 1.272 |  | 20 |  |
|  |  | MIXED |  | 3.500 |  | 1.654 |  | 18 |  |
|  |  | TD |  | 3.600 |  | 1.363 |  | 20 |  |
| comp |  | ASD |  | 3.017 |  | 1.259 |  | 20 |  |
|  |  | MIXED |  | 3.528 |  | 1.693 |  | 18 |  |
|  |  | TD |  | 3.500 |  | 1.386 |  | 20 |  |
| meal |  | ASD |  | 4.916 |  | 0.863 |  | 20 |  |
|  |  | MIXED |  | 4.639 |  | 1.513 |  | 18 |  |
|  |  | TD |  | 4.900 |  | 1.177 |  | 20 |  |
| roleplay |  | ASD |  | 2.879 |  | 1.694 |  | 20 |  |
|  |  | MIXED |  | 3.806 |  | 1.750 |  | 18 |  |
|  |  | TD |  | 4.050 |  | 1.547 |  | 20 |  |
|  | | | | | | | | | |

**Descriptives Plot**


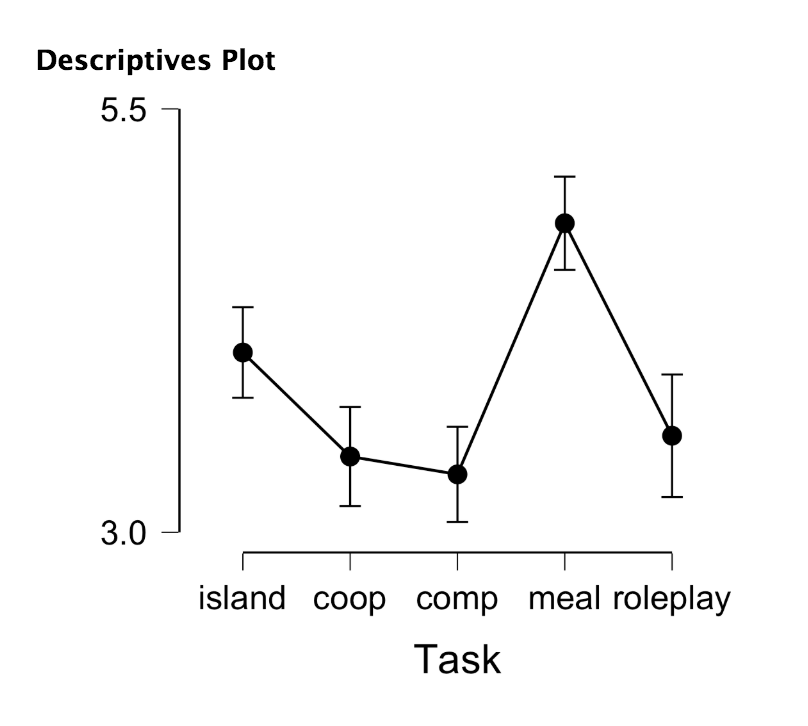


## How pleasant was the interaction?

Repeated Measures ANOVA

| **Within Subjects Effects** | | | | | | | | | | | | | | | |
| --- | --- | --- | --- | --- | --- | --- | --- | --- | --- | --- | --- | --- | --- | --- | --- |
|  | | **Sphericity Correction** | | **Sum of Squares** | | **df** | | **Mean Square** | | **F** | | **p** | | **η² _p_** | |
| Task |  | Greenhouse-Geisser |  | 72.683 | ᵃ | 2.914 | ᵃ | 24.946 | ᵃ | 23.481 | ᵃ | < .001 | ᵃ | 0.299 |  |
| Task ✻ group3 |  | Greenhouse-Geisser |  | 16.613 | ᵃ | 5.827 | ᵃ | 2.851 | ᵃ | 2.683 | ᵃ | 0.018 | ᵃ | 0.089 |  |
| Residual |  | Greenhouse-Geisser |  | 170.248 |  | 160.248 |  | 1.062 |  |  |  |  |  |  |  |
|  | | | | | | | | | | | | | | | |
| *Note.*  Type III Sum of Squares | | | | | | | | | | | | | | | |
| ᵃ Mauchly's test of sphericity indicates that the assumption of sphericity is violated (p < .05). | | | | | | | | | | | | | | | |

| **Between Subjects Effects** | | | | | | | | | | | | | |
| --- | --- | --- | --- | --- | --- | --- | --- | --- | --- | --- | --- | --- | --- |
|  | | **Sum of Squares** | | **df** | | **Mean Square** | | **F** | | **p** | | **η² _p_** | |
| group3 |  | 28.076 |  | 2 |  | 14.038 |  | 3.336 |  | 0.043 |  | 0.108 |  |
| Residual |  | 231.457 |  | 55 |  | 4.208 |  |  |  |  |  |  |  |
|  | | | | | | | | | | | | | |
| *Note.*  Type III Sum of Squares | | | | | | | | | | | | | |

Assumption Checks

| **Test of Sphericity** | | | | | | | | | | | | | | | |
| --- | --- | --- | --- | --- | --- | --- | --- | --- | --- | --- | --- | --- | --- | --- | --- |
|  | | **Mauchly's W** | | **Approx. Χ²** | | **df** | | **p** | | **Greenhouse-Geisser ε** | | **Huynh-Feldt ε** | | **Lower Bound ε** | |
| Task |  | 0.492 |  | 37.857 |  | 9 |  | < .001 |  | 0.728 |  | 0.774 |  | 0.250 |  |
|  | | | | | | | | | | | | | | | |

| **Test for Equality of Variances (Levene's)** | | | | | | | | | |
| --- | --- | --- | --- | --- | --- | --- | --- | --- | --- |
|  | | **F** | | **df1** | | **df2** | | **p** | |
| Insel_angenehm |  | 2.375 |  | 2.000 |  | 55.000 |  | 0.102 |  |
| Koop_angenehm |  | 1.685 |  | 2.000 |  | 55.000 |  | 0.195 |  |
| Kompet_angenehm |  | 0.187 |  | 2.000 |  | 55.000 |  | 0.830 |  |
| Meal_angenehm |  | 0.122 |  | 2.000 |  | 55.000 |  | 0.885 |  |
| Roll_angenehm |  | 0.521 |  | 2.000 |  | 55.000 |  | 0.597 |  |
|  | | | | | | | | | |

Post Hoc Tests

| **Post Hoc Comparisons - Task ✻ group3** | | | | | | | | | | | | | | | | | |
| --- | --- | --- | --- | --- | --- | --- | --- | --- | --- | --- | --- | --- | --- | --- | --- | --- | --- |
|  | | | | | | **95% CI of Mean Difference** | | | |  | | | | | | | |
|  | |  | | **Mean Difference** | | **Lower** | | **Upper** | | **SE** | | **t** | | **Cohen's d** | | **p _holm_** | |
| comp,ASD |  | comp,MIXED |  | -0.074 |  | -1.477 |  | 1.330 |  | 0.393 |  | -0.187 |  | -0.025 |  | 1.000 |  |
|  |  | comp,TD |  | -0.393 |  | -1.759 |  | 0.973 |  | 0.382 |  | -1.028 |  | -0.135 |  | 1.000 |  |
|  |  | coop,ASD |  | -0.081 |  | -1.068 |  | 0.906 |  | 0.278 |  | -0.292 |  | -0.038 |  | 1.000 |  |
|  |  | coop,MIXED |  | 0.176 |  | -1.227 |  | 1.580 |  | 0.393 |  | 0.449 |  | 0.059 |  | 1.000 |  |
|  |  | coop,TD |  | -0.893 |  | -2.259 |  | 0.473 |  | 0.382 |  | -2.336 |  | -0.307 |  | 1.000 |  |
|  |  | island,ASD |  | -0.142 |  | -1.128 |  | 0.845 |  | 0.278 |  | -0.509 |  | -0.067 |  | 1.000 |  |
|  |  | island,MIXED |  | -0.074 |  | -1.477 |  | 1.330 |  | 0.393 |  | -0.187 |  | -0.025 |  | 1.000 |  |
|  |  | island,TD |  | -1.218 |  | -2.584 |  | 0.148 |  | 0.382 |  | -3.187 |  | -0.418 |  | 0.132 |  |
|  |  | meal,ASD |  | -1.227 |  | -2.213 |  | -0.240 |  | 0.278 |  | -4.410 |  | -0.579 |  | 0.002 |  |
|  |  | meal,MIXED |  | -0.907 |  | -2.310 |  | 0.497 |  | 0.393 |  | -2.309 |  | -0.303 |  | 1.000 |  |
|  |  | meal,TD |  | -1.418 |  | -2.784 |  | -0.052 |  | 0.382 |  | -3.710 |  | -0.487 |  | 0.026 |  |
|  |  | roleplay,ASD |  | 1.001 |  | 0.015 |  | 1.988 |  | 0.278 |  | 3.599 |  | 0.473 |  | 0.034 |  |
|  |  | roleplay,MIXED |  | 0.038 |  | -1.366 |  | 1.441 |  | 0.393 |  | 0.096 |  | 0.013 |  | 1.000 |  |
|  |  | roleplay,TD |  | 0.032 |  | -1.334 |  | 1.398 |  | 0.382 |  | 0.084 |  | 0.011 |  | 1.000 |  |
| comp,MIXED |  | comp,TD |  | -0.319 |  | -1.723 |  | 1.084 |  | 0.393 |  | -0.814 |  | -0.107 |  | 1.000 |  |
|  |  | coop,ASD |  | -0.008 |  | -1.411 |  | 1.396 |  | 0.393 |  | -0.019 |  | -0.003 |  | 1.000 |  |
|  |  | coop,MIXED |  | 0.250 |  | -0.790 |  | 1.290 |  | 0.293 |  | 0.853 |  | 0.112 |  | 1.000 |  |
|  |  | coop,TD |  | -0.819 |  | -2.223 |  | 0.584 |  | 0.393 |  | -2.087 |  | -0.274 |  | 1.000 |  |
|  |  | island,ASD |  | -0.068 |  | -1.472 |  | 1.335 |  | 0.393 |  | -0.174 |  | -0.023 |  | 1.000 |  |
|  |  | island,MIXED |  | 1.651e -15 |  | -1.040 |  | 1.040 |  | 0.293 |  | 5.632e -15 |  | 7.395e -16 |  | 1.000 |  |
|  |  | island,TD |  | -1.144 |  | -2.548 |  | 0.259 |  | 0.393 |  | -2.915 |  | -0.383 |  | 0.289 |  |
|  |  | meal,ASD |  | -1.153 |  | -2.557 |  | 0.250 |  | 0.393 |  | -2.937 |  | -0.386 |  | 0.277 |  |
|  |  | meal,MIXED |  | -0.833 |  | -1.873 |  | 0.207 |  | 0.293 |  | -2.842 |  | -0.373 |  | 0.334 |  |
|  |  | meal,TD |  | -1.344 |  | -2.748 |  | 0.059 |  | 0.393 |  | -3.424 |  | -0.450 |  | 0.067 |  |
|  |  | roleplay,ASD |  | 1.075 |  | -0.329 |  | 2.478 |  | 0.393 |  | 2.737 |  | 0.359 |  | 0.453 |  |
|  |  | roleplay,MIXED |  | 0.111 |  | -0.929 |  | 1.151 |  | 0.293 |  | 0.379 |  | 0.050 |  | 1.000 |  |
|  |  | roleplay,TD |  | 0.106 |  | -1.298 |  | 1.509 |  | 0.393 |  | 0.269 |  | 0.035 |  | 1.000 |  |
| comp,TD |  | coop,ASD |  | 0.312 |  | -1.054 |  | 1.678 |  | 0.382 |  | 0.816 |  | 0.107 |  | 1.000 |  |
|  |  | coop,MIXED |  | 0.569 |  | -0.834 |  | 1.973 |  | 0.393 |  | 1.450 |  | 0.190 |  | 1.000 |  |
|  |  | coop,TD |  | -0.500 |  | -1.487 |  | 0.487 |  | 0.278 |  | -1.797 |  | -0.236 |  | 1.000 |  |
|  |  | island,ASD |  | 0.251 |  | -1.115 |  | 1.617 |  | 0.382 |  | 0.658 |  | 0.086 |  | 1.000 |  |
|  |  | island,MIXED |  | 0.319 |  | -1.084 |  | 1.723 |  | 0.393 |  | 0.814 |  | 0.107 |  | 1.000 |  |
|  |  | island,TD |  | -0.825 |  | -1.812 |  | 0.162 |  | 0.278 |  | -2.966 |  | -0.389 |  | 0.248 |  |
|  |  | meal,ASD |  | -0.834 |  | -2.200 |  | 0.532 |  | 0.382 |  | -2.182 |  | -0.286 |  | 1.000 |  |
|  |  | meal,MIXED |  | -0.514 |  | -1.917 |  | 0.890 |  | 0.393 |  | -1.309 |  | -0.172 |  | 1.000 |  |
|  |  | meal,TD |  | -1.025 |  | -2.012 |  | -0.038 |  | 0.278 |  | -3.685 |  | -0.484 |  | 0.026 |  |
|  |  | roleplay,ASD |  | 1.394 |  | 0.028 |  | 2.760 |  | 0.382 |  | 3.648 |  | 0.479 |  | 0.032 |  |
|  |  | roleplay,MIXED |  | 0.431 |  | -0.973 |  | 1.834 |  | 0.393 |  | 1.096 |  | 0.144 |  | 1.000 |  |
|  |  | roleplay,TD |  | 0.425 |  | -0.562 |  | 1.412 |  | 0.278 |  | 1.528 |  | 0.201 |  | 1.000 |  |
| coop,ASD |  | coop,MIXED |  | 0.258 |  | -1.146 |  | 1.661 |  | 0.393 |  | 0.656 |  | 0.086 |  | 1.000 |  |
|  |  | coop,TD |  | -0.812 |  | -2.178 |  | 0.554 |  | 0.382 |  | -2.124 |  | -0.279 |  | 1.000 |  |
|  |  | island,ASD |  | -0.061 |  | -1.047 |  | 0.926 |  | 0.278 |  | -0.218 |  | -0.029 |  | 1.000 |  |
|  |  | island,MIXED |  | 0.008 |  | -1.396 |  | 1.411 |  | 0.393 |  | 0.019 |  | 0.003 |  | 1.000 |  |
|  |  | island,TD |  | -1.137 |  | -2.503 |  | 0.229 |  | 0.382 |  | -2.974 |  | -0.391 |  | 0.251 |  |
|  |  | meal,ASD |  | -1.146 |  | -2.132 |  | -0.159 |  | 0.278 |  | -4.118 |  | -0.541 |  | 0.005 |  |
|  |  | meal,MIXED |  | -0.826 |  | -2.229 |  | 0.578 |  | 0.393 |  | -2.103 |  | -0.276 |  | 1.000 |  |
|  |  | meal,TD |  | -1.337 |  | -2.703 |  | 0.029 |  | 0.382 |  | -3.498 |  | -0.459 |  | 0.052 |  |
|  |  | roleplay,ASD |  | 1.082 |  | 0.096 |  | 2.069 |  | 0.278 |  | 3.891 |  | 0.511 |  | 0.012 |  |
|  |  | roleplay,MIXED |  | 0.119 |  | -1.285 |  | 1.522 |  | 0.393 |  | 0.302 |  | 0.040 |  | 1.000 |  |
|  |  | roleplay,TD |  | 0.113 |  | -1.253 |  | 1.479 |  | 0.382 |  | 0.296 |  | 0.039 |  | 1.000 |  |
| coop,MIXED |  | coop,TD |  | -1.069 |  | -2.473 |  | 0.334 |  | 0.393 |  | -2.724 |  | -0.358 |  | 0.457 |  |
|  |  | island,ASD |  | -0.318 |  | -1.722 |  | 1.085 |  | 0.393 |  | -0.810 |  | -0.106 |  | 1.000 |  |
|  |  | island,MIXED |  | -0.250 |  | -1.290 |  | 0.790 |  | 0.293 |  | -0.853 |  | -0.112 |  | 1.000 |  |
|  |  | island,TD |  | -1.394 |  | -2.798 |  | 0.009 |  | 0.393 |  | -3.551 |  | -0.466 |  | 0.044 |  |
|  |  | meal,ASD |  | -1.403 |  | -2.807 |  | 3.791e -4 |  | 0.393 |  | -3.574 |  | -0.469 |  | 0.041 |  |
|  |  | meal,MIXED |  | -1.083 |  | -2.123 |  | -0.043 |  | 0.293 |  | -3.694 |  | -0.485 |  | 0.026 |  |
|  |  | meal,TD |  | -1.594 |  | -2.998 |  | -0.191 |  | 0.393 |  | -4.061 |  | -0.533 |  | 0.008 |  |
|  |  | roleplay,ASD |  | 0.825 |  | -0.579 |  | 2.228 |  | 0.393 |  | 2.101 |  | 0.276 |  | 1.000 |  |
|  |  | roleplay,MIXED |  | -0.139 |  | -1.179 |  | 0.901 |  | 0.293 |  | -0.474 |  | -0.062 |  | 1.000 |  |
|  |  | roleplay,TD |  | -0.144 |  | -1.548 |  | 1.259 |  | 0.393 |  | -0.368 |  | -0.048 |  | 1.000 |  |
| coop,TD |  | island,ASD |  | 0.751 |  | -0.615 |  | 2.117 |  | 0.382 |  | 1.966 |  | 0.258 |  | 1.000 |  |
|  |  | island,MIXED |  | 0.819 |  | -0.584 |  | 2.223 |  | 0.393 |  | 2.087 |  | 0.274 |  | 1.000 |  |
|  |  | island,TD |  | -0.325 |  | -1.312 |  | 0.662 |  | 0.278 |  | -1.168 |  | -0.153 |  | 1.000 |  |
|  |  | meal,ASD |  | -0.334 |  | -1.700 |  | 1.032 |  | 0.382 |  | -0.873 |  | -0.115 |  | 1.000 |  |
|  |  | meal,MIXED |  | -0.014 |  | -1.417 |  | 1.390 |  | 0.393 |  | -0.035 |  | -0.005 |  | 1.000 |  |
|  |  | meal,TD |  | -0.525 |  | -1.512 |  | 0.462 |  | 0.278 |  | -1.887 |  | -0.248 |  | 1.000 |  |
|  |  | roleplay,ASD |  | 1.894 |  | 0.528 |  | 3.260 |  | 0.382 |  | 4.956 |  | 0.651 |  | < .001 |  |
|  |  | roleplay,MIXED |  | 0.931 |  | -0.473 |  | 2.334 |  | 0.393 |  | 2.370 |  | 0.311 |  | 1.000 |  |
|  |  | roleplay,TD |  | 0.925 |  | -0.062 |  | 1.912 |  | 0.278 |  | 3.325 |  | 0.437 |  | 0.084 |  |
| island,ASD |  | island,MIXED |  | 0.068 |  | -1.335 |  | 1.472 |  | 0.393 |  | 0.174 |  | 0.023 |  | 1.000 |  |
|  |  | island,TD |  | -1.076 |  | -2.442 |  | 0.290 |  | 0.382 |  | -2.816 |  | -0.370 |  | 0.365 |  |
|  |  | meal,ASD |  | -1.085 |  | -2.072 |  | -0.098 |  | 0.278 |  | -3.901 |  | -0.512 |  | 0.012 |  |
|  |  | meal,MIXED |  | -0.765 |  | -2.169 |  | 0.638 |  | 0.393 |  | -1.949 |  | -0.256 |  | 1.000 |  |
|  |  | meal,TD |  | -1.276 |  | -2.642 |  | 0.090 |  | 0.382 |  | -3.339 |  | -0.438 |  | 0.085 |  |
|  |  | roleplay,ASD |  | 1.143 |  | 0.156 |  | 2.130 |  | 0.278 |  | 4.109 |  | 0.540 |  | 0.005 |  |
|  |  | roleplay,MIXED |  | 0.179 |  | -1.224 |  | 1.583 |  | 0.393 |  | 0.456 |  | 0.060 |  | 1.000 |  |
|  |  | roleplay,TD |  | 0.174 |  | -1.192 |  | 1.540 |  | 0.382 |  | 0.454 |  | 0.060 |  | 1.000 |  |
| island,MIXED |  | island,TD |  | -1.144 |  | -2.548 |  | 0.259 |  | 0.393 |  | -2.915 |  | -0.383 |  | 0.289 |  |
|  |  | meal,ASD |  | -1.153 |  | -2.557 |  | 0.250 |  | 0.393 |  | -2.937 |  | -0.386 |  | 0.277 |  |
|  |  | meal,MIXED |  | -0.833 |  | -1.873 |  | 0.207 |  | 0.293 |  | -2.842 |  | -0.373 |  | 0.334 |  |
|  |  | meal,TD |  | -1.344 |  | -2.748 |  | 0.059 |  | 0.393 |  | -3.424 |  | -0.450 |  | 0.067 |  |
|  |  | roleplay,ASD |  | 1.075 |  | -0.329 |  | 2.478 |  | 0.393 |  | 2.737 |  | 0.359 |  | 0.453 |  |
|  |  | roleplay,MIXED |  | 0.111 |  | -0.929 |  | 1.151 |  | 0.293 |  | 0.379 |  | 0.050 |  | 1.000 |  |
|  |  | roleplay,TD |  | 0.106 |  | -1.298 |  | 1.509 |  | 0.393 |  | 0.269 |  | 0.035 |  | 1.000 |  |
| island,TD |  | meal,ASD |  | -0.009 |  | -1.375 |  | 1.357 |  | 0.382 |  | -0.023 |  | -0.003 |  | 1.000 |  |
|  |  | meal,MIXED |  | 0.311 |  | -1.092 |  | 1.715 |  | 0.393 |  | 0.792 |  | 0.104 |  | 1.000 |  |
|  |  | meal,TD |  | -0.200 |  | -1.187 |  | 0.787 |  | 0.278 |  | -0.719 |  | -0.094 |  | 1.000 |  |
|  |  | roleplay,ASD |  | 2.219 |  | 0.853 |  | 3.585 |  | 0.382 |  | 5.807 |  | 0.762 |  | < .001 |  |
|  |  | roleplay,MIXED |  | 1.256 |  | -0.148 |  | 2.659 |  | 0.393 |  | 3.197 |  | 0.420 |  | 0.129 |  |
|  |  | roleplay,TD |  | 1.250 |  | 0.263 |  | 2.237 |  | 0.278 |  | 4.493 |  | 0.590 |  | 0.001 |  |
| meal,ASD |  | meal,MIXED |  | 0.320 |  | -1.084 |  | 1.723 |  | 0.393 |  | 0.815 |  | 0.107 |  | 1.000 |  |
|  |  | meal,TD |  | -0.191 |  | -1.557 |  | 1.175 |  | 0.382 |  | -0.500 |  | -0.066 |  | 1.000 |  |
|  |  | roleplay,ASD |  | 2.228 |  | 1.241 |  | 3.215 |  | 0.278 |  | 8.009 |  | 1.052 |  | < .001 |  |
|  |  | roleplay,MIXED |  | 1.264 |  | -0.139 |  | 2.668 |  | 0.393 |  | 3.220 |  | 0.423 |  | 0.122 |  |
|  |  | roleplay,TD |  | 1.259 |  | -0.107 |  | 2.625 |  | 0.382 |  | 3.294 |  | 0.432 |  | 0.098 |  |
| meal,MIXED |  | meal,TD |  | -0.511 |  | -1.915 |  | 0.892 |  | 0.393 |  | -1.302 |  | -0.171 |  | 1.000 |  |
|  |  | roleplay,ASD |  | 1.908 |  | 0.505 |  | 3.312 |  | 0.393 |  | 4.860 |  | 0.638 |  | < .001 |  |
|  |  | roleplay,MIXED |  | 0.944 |  | -0.096 |  | 1.985 |  | 0.293 |  | 3.221 |  | 0.423 |  | 0.115 |  |
|  |  | roleplay,TD |  | 0.939 |  | -0.465 |  | 2.342 |  | 0.393 |  | 2.391 |  | 0.314 |  | 1.000 |  |
| meal,TD |  | roleplay,ASD |  | 2.419 |  | 1.053 |  | 3.785 |  | 0.382 |  | 6.330 |  | 0.831 |  | < .001 |  |
|  |  | roleplay,MIXED |  | 1.456 |  | 0.052 |  | 2.859 |  | 0.393 |  | 3.707 |  | 0.487 |  | 0.026 |  |
|  |  | roleplay,TD |  | 1.450 |  | 0.463 |  | 2.437 |  | 0.278 |  | 5.212 |  | 0.684 |  | < .001 |  |
| roleplay,ASD |  | roleplay,MIXED |  | -0.964 |  | -2.367 |  | 0.440 |  | 0.393 |  | -2.454 |  | -0.322 |  | 0.933 |  |
|  |  | roleplay,TD |  | -0.969 |  | -2.335 |  | 0.397 |  | 0.382 |  | -2.536 |  | -0.333 |  | 0.760 |  |
| roleplay,MIXED |  | roleplay,TD |  | -0.006 |  | -1.409 |  | 1.398 |  | 0.393 |  | -0.014 |  | -0.002 |  | 1.000 |  |
|  | | | | | | | | | | | | | | | | | |
| *Note.*  Cohen's d does not correct for multiple comparisons. | | | | | | | | | | | | | | | | | |
| *Note.*  Bonferroni adjusted confidence intervals. | | | | | | | | | | | | | | | | | |

| Descriptives | | | | | | | | | |
| --- | --- | --- | --- | --- | --- | --- | --- | --- | --- |
| **Task** | | **group3** | | **Mean** | | **SD** | | **N** | |
| island |  | ASD |  | 4.124 |  | 1.190 |  | 20 |  |
|  |  | MIXED |  | 4.056 |  | 1.371 |  | 18 |  |
|  |  | TD |  | 5.200 |  | 0.750 |  | 20 |  |
| coop |  | ASD |  | 4.063 |  | 1.038 |  | 20 |  |
|  |  | MIXED |  | 3.806 |  | 1.457 |  | 18 |  |
|  |  | TD |  | 4.875 |  | 0.887 |  | 20 |  |
| comp |  | ASD |  | 3.982 |  | 1.187 |  | 20 |  |
|  |  | MIXED |  | 4.056 |  | 1.187 |  | 18 |  |
|  |  | TD |  | 4.375 |  | 1.157 |  | 20 |  |
| meal |  | ASD |  | 5.209 |  | 0.991 |  | 20 |  |
|  |  | MIXED |  | 4.889 |  | 1.267 |  | 18 |  |
|  |  | TD |  | 5.400 |  | 0.897 |  | 20 |  |
| roleplay |  | ASD |  | 2.981 |  | 1.554 |  | 20 |  |
|  |  | MIXED |  | 3.944 |  | 1.381 |  | 18 |  |
|  |  | TD |  | 3.950 |  | 1.538 |  | 20 |  |
|  | | | | | | | | | |

Descriptives Plot

 
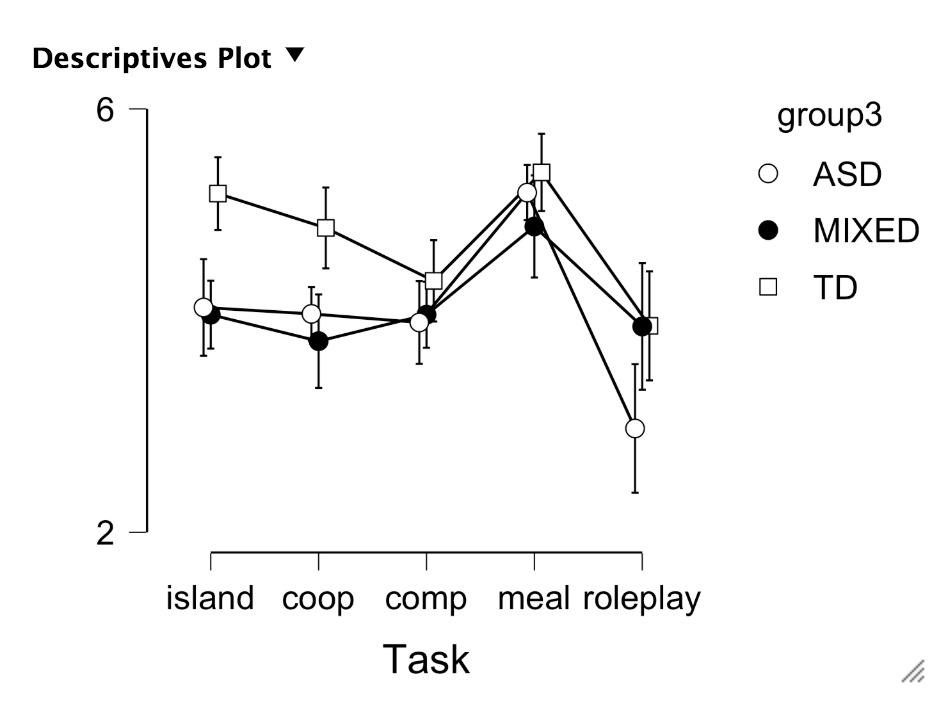


## How likeable was the partner?

Repeated Measures ANOVA

| **Within Subjects Effects** | | | | | | | | | | | | | | | |
| --- | --- | --- | --- | --- | --- | --- | --- | --- | --- | --- | --- | --- | --- | --- | --- |
|  | | **Sphericity Correction** | | **Sum of Squares** | | **df** | | **Mean Square** | | **F** | | **p** | | **η² _p_** | |
| Task |  | Greenhouse-Geisser |  | 14.088 | ᵃ | 2.160 | ᵃ | 6.523 | ᵃ | 6.538 | ᵃ | 0.002 | ᵃ | 0.106 |  |
| Task ✻ group3 |  | Greenhouse-Geisser |  | 3.952 | ᵃ | 4.320 | ᵃ | 0.915 | ᵃ | 0.917 | ᵃ | 0.462 | ᵃ | 0.032 |  |
| Residual |  | Greenhouse-Geisser |  | 118.516 |  | 118.787 |  | 0.998 |  |  |  |  |  |  |  |
|  | | | | | | | | | | | | | | | |
| *Note.*  Type III Sum of Squares | | | | | | | | | | | | | | | |
| ᵃ Mauchly's test of sphericity indicates that the assumption of sphericity is violated (p < .05). | | | | | | | | | | | | | | | |

| **Between Subjects Effects** | | | | | | | | | | | | | |
| --- | --- | --- | --- | --- | --- | --- | --- | --- | --- | --- | --- | --- | --- |
|  | | **Sum of Squares** | | **df** | | **Mean Square** | | **F** | | **p** | | **η² _p_** | |
| group3 |  | 1622.837 |  | 2 |  | 811.419 |  | 1.061 |  | 0.353 |  | 0.037 |  |
| Residual |  | 42066.238 |  | 55 |  | 764.841 |  |  |  |  |  |  |  |
|  | | | | | | | | | | | | | |
| *Note.*  Type III Sum of Squares | | | | | | | | | | | | | |

Assumption Checks

| **Test of Sphericity** | | | | | | | | | | | | | | | |
| --- | --- | --- | --- | --- | --- | --- | --- | --- | --- | --- | --- | --- | --- | --- | --- |
|  | | **Mauchly's W** | | **Approx. Χ²** | | **df** | | **p** | | **Greenhouse-Geisser ε** | | **Huynh-Feldt ε** | | **Lower Bound ε** | |
| Task |  | 0.170 |  | 94.793 |  | 9 |  | < .001 |  | 0.540 |  | 0.563 |  | 0.250 |  |
|  | | | | | | | | | | | | | | | |

| **Test for Equality of Variances (Levene's)** | | | | | | | | | |
| --- | --- | --- | --- | --- | --- | --- | --- | --- | --- |
|  | | **F** | | **df1** | | **df2** | | **p** | |
| Insel_sympathis |  | 4.195 |  | 2.000 |  | 55.000 |  | 0.020 |  |
| Koop_sympathisch |  | 4.308 |  | 2.000 |  | 55.000 |  | 0.018 |  |
| Kompet_sympathisch |  | 4.169 |  | 2.000 |  | 55.000 |  | 0.021 |  |
| Meal_unsympathisch |  | 4.265 |  | 2.000 |  | 55.000 |  | 0.019 |  |
| Roll_sympathisch |  | 3.902 |  | 2.000 |  | 55.000 |  | 0.026 |  |
|  | | | | | | | | | |

Post Hoc Tests

| **Post Hoc Comparisons - Task** | | | | | | | | | | | | | | | | | |
| --- | --- | --- | --- | --- | --- | --- | --- | --- | --- | --- | --- | --- | --- | --- | --- | --- | --- |
|  | | | | | | **95% CI of Mean Difference** | | | |  | | | | | | | |
|  | |  | | **Mean Difference** | | **Lower** | | **Upper** | | **SE** | | **t** | | **Cohen's d** | | **p _holm_** | |
| comp |  | coop |  | -0.202 |  | -0.519 |  | 0.115 |  | 0.109 |  | -1.859 |  | -0.244 |  | 0.349 |  |
|  |  | island |  | -0.211 |  | -0.596 |  | 0.175 |  | 0.132 |  | -1.597 |  | -0.210 |  | 0.349 |  |
|  |  | meal |  | -0.518 |  | -0.809 |  | -0.226 |  | 0.100 |  | -5.182 |  | -0.680 |  | < .001 |  |
|  |  | roleplay |  | 0.140 |  | -0.337 |  | 0.618 |  | 0.164 |  | 0.858 |  | 0.113 |  | 0.789 |  |
| coop |  | island |  | -0.009 |  | -0.234 |  | 0.217 |  | 0.077 |  | -0.114 |  | -0.015 |  | 0.910 |  |
|  |  | meal |  | -0.316 |  | -0.521 |  | -0.111 |  | 0.070 |  | -4.495 |  | -0.590 |  | < .001 |  |
|  |  | roleplay |  | 0.342 |  | -0.175 |  | 0.859 |  | 0.177 |  | 1.933 |  | 0.254 |  | 0.349 |  |
| island |  | meal |  | -0.307 |  | -0.573 |  | -0.041 |  | 0.091 |  | -3.374 |  | -0.443 |  | 0.009 |  |
|  |  | roleplay |  | 0.351 |  | -0.208 |  | 0.910 |  | 0.191 |  | 1.834 |  | 0.241 |  | 0.349 |  |
| meal |  | roleplay |  | 0.658 |  | 0.134 |  | 1.182 |  | 0.180 |  | 3.664 |  | 0.481 |  | 0.004 |  |
|  | | | | | | | | | | | | | | | | | |
| *Note.*  Cohen's d does not correct for multiple comparisons. | | | | | | | | | | | | | | | | | |
| *Note.*  Bonferroni adjusted confidence intervals. | | | | | | | | | | | | | | | | | |

| Descriptives | | | | | | | | | |
| --- | --- | --- | --- | --- | --- | --- | --- | --- | --- |
| **Task** | | **group3** | | **Mean** | | **SD** | | **N** | |
| island |  | ASD |  | 4.856 |  | 1.580 |  | 20 |  |
|  |  | MIXED |  | 10.083 |  | 22.210 |  | 18 |  |
|  |  | TD |  | 5.250 |  | 0.698 |  | 20 |  |
| coop |  | ASD |  | 4.730 |  | 1.407 |  | 20 |  |
|  |  | MIXED |  | 10.028 |  | 22.222 |  | 18 |  |
|  |  | TD |  | 5.400 |  | 0.476 |  | 20 |  |
| comp |  | ASD |  | 4.720 |  | 1.431 |  | 20 |  |
|  |  | MIXED |  | 9.944 |  | 22.237 |  | 18 |  |
|  |  | TD |  | 4.900 |  | 0.926 |  | 20 |  |
| meal |  | ASD |  | 5.221 |  | 1.423 |  | 20 |  |
|  |  | MIXED |  | 10.417 |  | 22.116 |  | 18 |  |
|  |  | TD |  | 5.475 |  | 0.595 |  | 20 |  |
| roleplay |  | ASD |  | 4.638 |  | 1.446 |  | 20 |  |
|  |  | MIXED |  | 9.944 |  | 22.242 |  | 18 |  |
|  |  | TD |  | 4.575 |  | 1.331 |  | 20 |  |
|  | | | | | | | | | |

Descriptives Plot


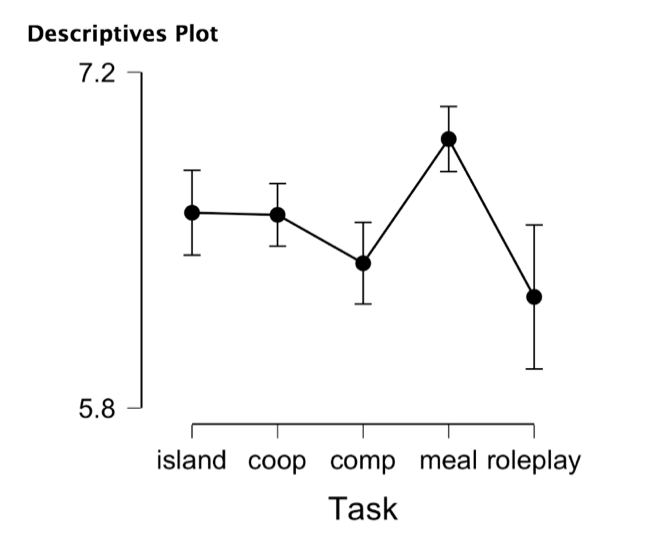


## Correlations between IPS and evaluation ratings:


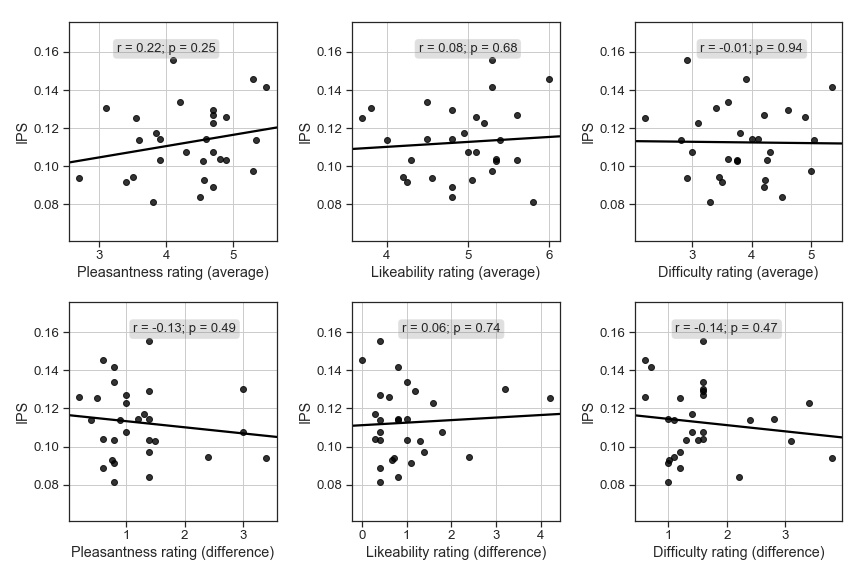

Supplement: Supplementary file 1 — Additional file 1. Supplementary Materials – Synchrony (IPS). Supplementary Materials – Motion Energy. Supplementary Materials – Evaluation measures [file 13229_2019_305_MOESM1_ESM.zip › Supplementary_analysis_evaluation.docx]
