# Supplementary material for: Reduced nonverbal interpersonal synchrony in autism spectrum disorder independent of partner diagnosis: a motion energy study
Source: Mol Autism. 2020 Feb 3;11:11. doi: 10.1186/s13229-019-0305-1 (PMC6998161; doi:10.1186/s13229-019-0305-1)
Supplement: Supplementary file 1 — Additional file 1. Supplementary Materials – Synchrony (IPS). Supplementary Materials – Motion Energy. Supplementary Materials – Evaluation measures [file 13229_2019_305_MOESM1_ESM.zip › Supplementary_analysis_motion_energy.pdf]

MOTION ENERGY results

Within Subjects Effects

	Sphericity Correction	Sum of Squares	df	Mean Square	F	p	η²	η² p
ROI	Greenhouse-Geisser	1.838e +6	1.000	1.838e +6	112.921	< .001	0.226	0.672
ROI ✻ diagnosis_group	Greenhouse-Geisser	25457.483	2.000	12728.742	0.782	0.463	0.003	0.028
Residual	Greenhouse-Geisser	895396.065	55.000	16279.928				
TASK	Greenhouse-Geisser	62058.907 [a]	3.121 [a]	19882.724 [a]	3.926 [a]	0.009 [a]	0.008	0.067
TASK ✻ diagnosis_group	Greenhouse-Geisser	29525.854 [a]	6.242 [a]	4729.816 [a]	0.934 [a]	0.475 [a]	0.004	0.033
Residual	Greenhouse-Geisser	869445.078	171.669	5064.671				
ROI ✻ TASK	Greenhouse-Geisser	20704.594	3.832	5403.134	3.360	0.012	0.003	0.058
ROI ✻ TASK ✻ diagnosis_group	Greenhouse-Geisser	8321.080	7.664	1085.747	0.675	0.707	0.001	0.024
Residual	Greenhouse-Geisser	338864.767	210.758	1607.840				

Note. Type III Sum of Squares

[a] Mauchly's test of sphericity indicates that the assumption of sphericity is violated ($p < .05$).

Between Subjects Effects

	Sum of Squares	df	Mean Square	F	p	η²	η² p
diagnosis_group	334697.968	2	167348.984	2.477	0.093	0.083	0.083
Residual	3.716e +6	55	67564.338				

Note. Type III Sum of Squares

Assumption Checks

Test of Sphericity

	Mauchly's W	Approx. X^2	df	p	Greenhouse-Geisser ε	Huynh-Feldt ε	Lower Bound ε
ROI	1.000 [a]	NaN [a]	NaN [a]	NaN [a]	1.000 [a]	1.000 [a]	1.000 [a]
TASK	0.607	26.694	9	0.002	0.780	0.833	0.250
ROI ✻ TASK	0.919	4.494	9	0.876	0.958	1.000	0.250

[a] Singular error SSP matrix: The repeated measure has only two levels, or more levels than observations. When the repeated measure has two levels, the assumption of sphericity is always met.

Descriptives

ROI	TASK	diagnosis_group	Mean	SD	N
Head	island	ASD	48.999	39.368	20
		TD	68.424	44.371	20
		mixed	33.322	25.129	18
	debate_coop	ASD	57.979	66.742	20
		TD	71.220	48.947	20
		mixed	27.133	21.972	18
	debate_comp	ASD	55.073	68.239	20
		TD	76.536	63.391	20
		mixed	33.421	27.622	18
	meal_planning	ASD	73.131	67.500	20
		TD	101.619	65.255	20
		mixed	44.823	26.402	18
	roleplay	ASD	73.495	90.623	20
		TD	79.386	57.583	20
		mixed	40.077	43.041	18
Body	island	ASD	158.853	115.453	20
		TD	176.941	107.535	20
		mixed	112.531	67.642	18
	debate_coop	ASD	199.572	182.402	20
		TD	196.411	137.057	20
		mixed	129.992	79.746	18
	debate_comp	ASD	182.206	161.702	20
		TD	218.884	191.938	20
		mixed	141.076	101.362	18
	meal_planning	ASD	183.821	137.791	20
		TD	241.175	146.033	20
		mixed	148.299	75.734	18
	roleplay	ASD	190.265	173.983	20
		TD	180.362	131.855	20
		mixed	115.301	110.590	18

Descriptives Plot

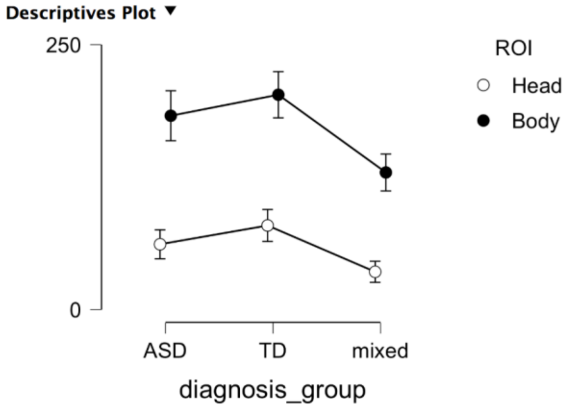

Descriptives Plots – Task & ROI

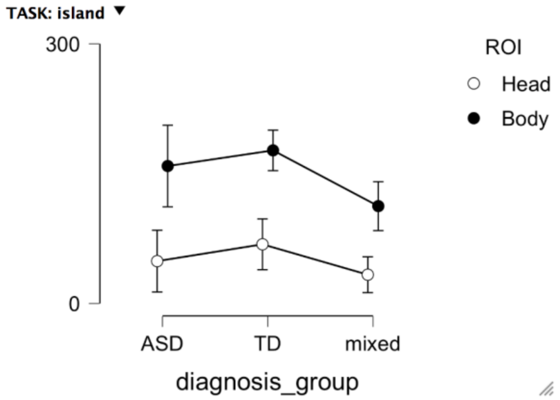

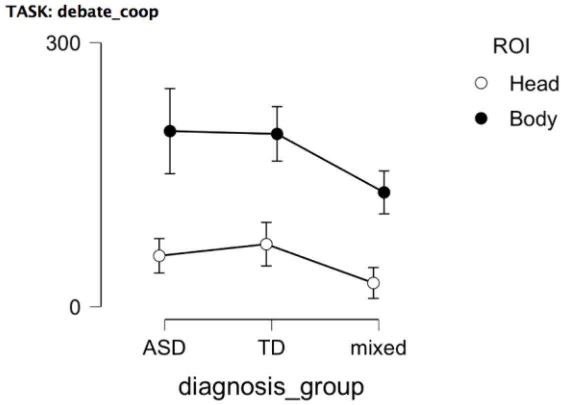

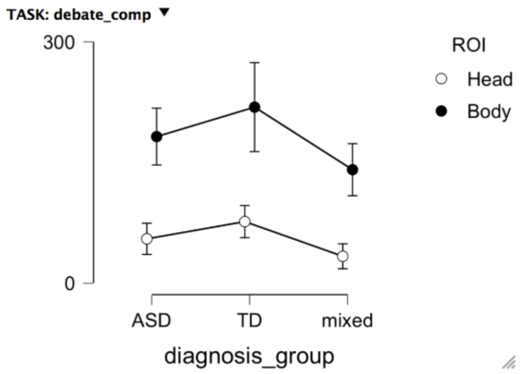
TASK: debate_comp
300
0
ROI
Head
Body
ASD
TD
mixed
diagnosis_group

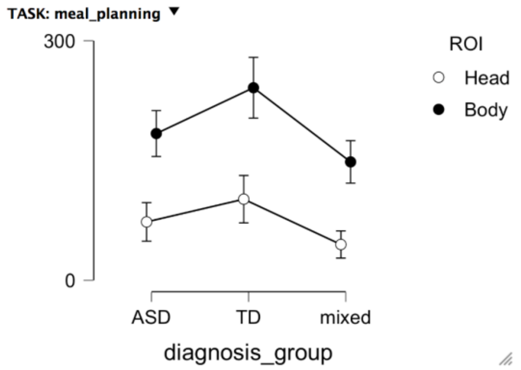
TASK: meal_planning
300
0
ROI
Head
Body
ASD
TD
mixed
diagnosis_group

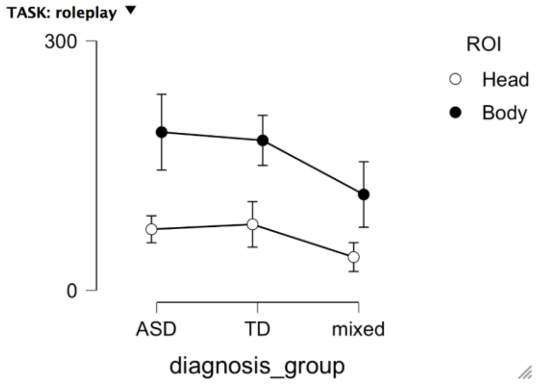
TASK: roleplay
300
0
ROI
Head
Body
ASD
TD
mixed
diagnosis_group
