# Supplementary material for: Reduced nonverbal interpersonal synchrony in autism spectrum disorder independent of partner diagnosis: a motion energy study
Source: Mol Autism. 2020 Feb 3;11:11. doi: 10.1186/s13229-019-0305-1 (PMC6998161; doi:10.1186/s13229-019-0305-1)
Supplement: Supplementary file 1 — Additional file 1. Supplementary Materials – Synchrony (IPS). Supplementary Materials – Motion Energy. Supplementary Materials – Evaluation measures [file 13229_2019_305_MOESM1_ESM.zip › Supplementary_analysis_synchrony.pdf]

IPS/SYNCHRONY results

Repeated Measures ANOVA

Within Subjects Effects

	Sphericity Correction	Sum of Squares	df	Mean Square	F	p	η²
ROI	Greenhouse-Geisser	0.014	1.000	0.014	44.832	< .001	0.071
ROI ✻ diagnosis_group	Greenhouse-Geisser	0.001	2.000	6.390e -4	2.103	0.142	0.007
Residual	Greenhouse-Geisser	0.008	26.000	3.038e -4			
Task	Greenhouse-Geisser	0.012 [a]	2.925 [a]	0.004 [a]	6.628 [a]	< .001 [a]	0.063
Task ✻ diagnosis_group	Greenhouse-Geisser	0.001 [a]	5.851 [a]	2.535e -4 [a]	0.408 [a]	0.868 [a]	0.008
Residual	Greenhouse-Geisser	0.047	76.058	6.220e -4			
ROI ✻ Task	Greenhouse-Geisser	8.895e -4	3.255	2.733e -4	1.613	0.189	0.005
ROI ✻ Task ✻ diagnosis_group	Greenhouse-Geisser	0.001	6.510	1.764e -4	1.041	0.407	0.006
Residual	Greenhouse-Geisser	0.014	84.627	1.694e -4			

Note. Type III Sum of Squares

[a] Mauchly's test of sphericity indicates that the assumption of sphericity is violated (p < .05).

Between Subjects Effects

	Sum of Squares	df	Mean Square	F	p	η²
diagnosis_group	0.022	2	0.011	4.041	0.030	0.237
Residual	0.069	26	0.003			

Note. Type III Sum of Squares

Assumption Checks

Test of Sphericity

	Mauchly's W	Approx. X^2	df	p	Greenhouse-Geisser ε	Huynh-Feldt ε	Lower Bound ε
ROI	1.000 [a]	NaN [a]	NaN [a]	NaN [a]	1.000 [a]	1.000 [a]	1.000 [a]
Task	0.418	21.295	9	0.012	0.731	0.834	0.250
ROI ✻ Task	0.624	11.528	9	0.242	0.814	0.944	0.250

[a] Singular error SSP matrix: The repeated measure has only two levels, or more levels than observations. When the repeated measure has two levels, the assumption of sphericity is always met.

Descriptives

ROI	Task	diagnosis_group	Mean	SD	N
Head	island	ASD	0.077	0.022	10
		TD	0.100	0.028	10
		mixed	0.077	0.012	9
	debate_coop	ASD	0.077	0.012	10
		TD	0.094	0.015	10
		mixed	0.074	0.014	9
	debate_comp	ASD	0.071	0.019	10
		TD	0.096	0.024	10
		mixed	0.079	0.026	9
	meal_planning	ASD	0.095	0.026	10
		TD	0.121	0.030	10
		mixed	0.092	0.020	9
	roleplay	ASD	0.084	0.026	10
		TD	0.097	0.024	10
		mixed	0.086	0.030	9
Body	island	ASD	0.091	0.014	10
		TD	0.104	0.017	10
		mixed	0.096	0.018	9
	debate_coop	ASD	0.098	0.023	10
		TD	0.109	0.029	10
		mixed	0.090	0.017	9
	debate_comp	ASD	0.098	0.018	10
		TD	0.114	0.029	10
		mixed	0.089	0.018	9
	meal_planning	ASD	0.114	0.030	10
		TD	0.124	0.023	10
		mixed	0.099	0.019	9
	roleplay	ASD	0.100	0.028	10
		TD	0.109	0.035	10
		mixed	0.091	0.018	9

Descriptives Plot - ROI

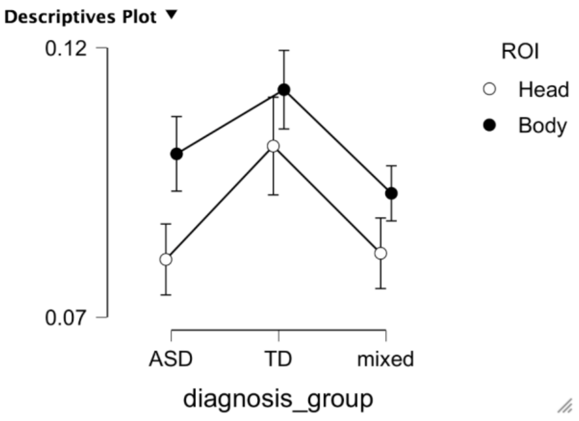

Descriptives Plots – Task & ROI

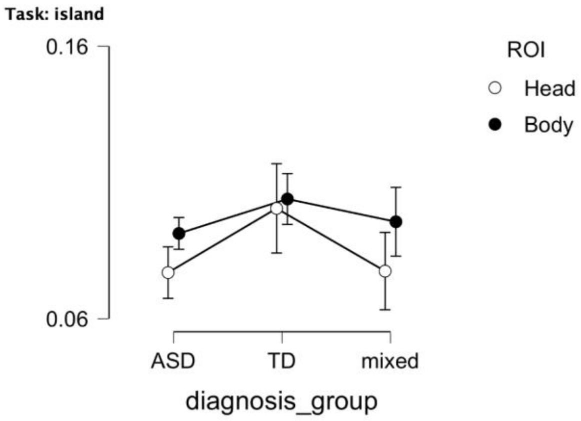

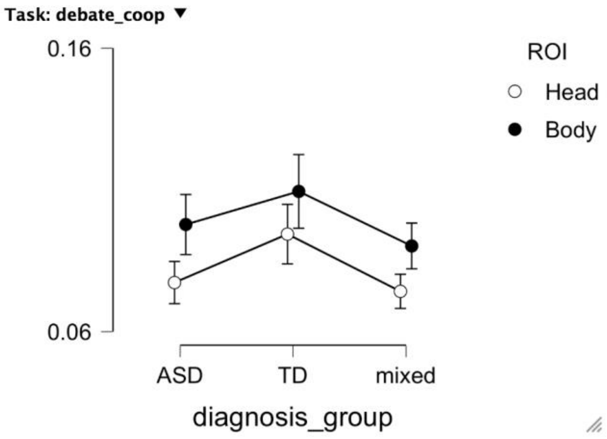

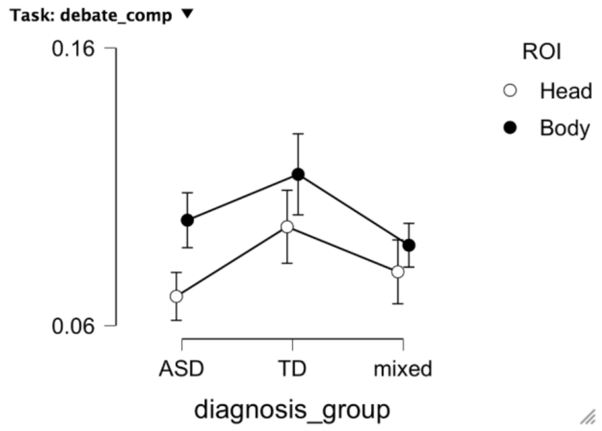
Task: debate_comp
0.16
0.06
ASD
TD
mixed
diagnosis_group
ROI
Head
Body

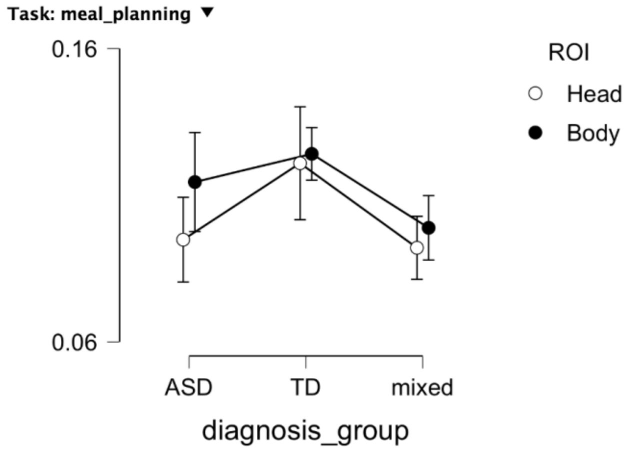
Task: meal_planning
0.16
0.06
ASD
TD
mixed
diagnosis_group
ROI
Head
Body

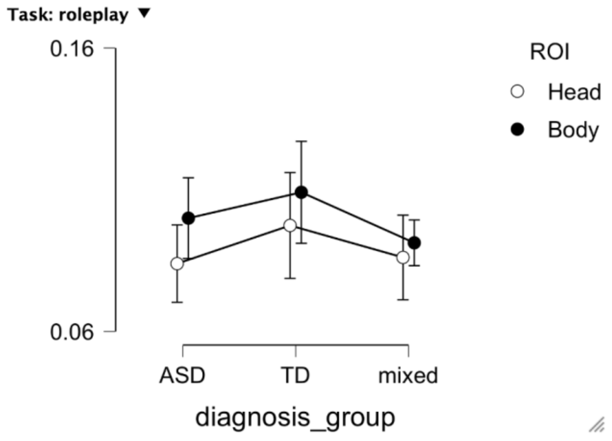
Task: roleplay
0.16
0.06
ASD
TD
mixed
diagnosis_group
ROI
Head
Body
